# The role of spatial and spatial-temporal analysis in children’s causal cognition of continuous processes

**DOI:** 10.1371/journal.pone.0235884

**Published:** 2020-07-30

**Authors:** Selma Dündar-Coecke, Andrew Tolmie, Anne Schlottmann

**Affiliations:** 1 Centre for Educational Neuroscience and Department of Psychology and Human Development, UCL Institute of Education, University College London, London, United Kingdom; 2 Department of Experimental Psychology, University College London, London, United Kingdom; University of Geneva, SWITZERLAND

## Abstract

Past research has largely ignored children’s ability to conjointly manipulate spatial and temporal information, but there are indications that the capacity to do so may provide important support for reasoning about causal processes. We hypothesised that spatial-temporal thinking is central to children’s ability to identify the invisible mechanisms that tie cause and effect together in continuous casual processes, which are focal in primary school science and crucial to understanding of the natural world. We investigated this in two studies (N = 107, N = 124), employing two methodologies, one shorter, the other more in depth. Further tasks assessed spatial-temporal (flow of liquid, extrapolation of relative speed, distance-time-velocity), spatial (two mental rotation, paper folding), verbal (expressive vocabulary), and nonverbal (block design) ability. Age dependent patterns were detected for both causal and predictor tasks. Two spatial-temporal tasks were unique and central predictors of children’s causal reasoning, especially inference of mechanism. Nonverbal ability predicted the simpler components of causal reasoning. One mental rotation task predicted only young children’s causal thinking. Verbal ability became significant when the sample included children from a wide range of socioeconomic backgrounds. Causal reasoning about continuous processes, including inferences of causal mechanism, appears to be within the reach of children from school entry age, but mechanism inference is uncommon. Analytic forms of spatial-temporal capacity seem to be important requirements for children to progress to this rather than merely perceptual forms.

## 1. Introduction

Although they have clear points of connection, children’s spatial and temporal cognition have typically been investigated separately, with limited consideration of how they might combine, as for example in visualising the trajectory of a ball over continuous moments after it has been thrown (see e.g. [1–5], for work on spatial thinking; and [6–9], for work on cognition of time). The present study postulates that most spatial qualities, such as nearness, distance, length, cannot be conceived independent of time, and vice versa. Therefore the aim is (1) to examine the nature and development of *spatial-temporal* cognition as an unified ability, and (2) to elaborate whether spatial-temporal cognition has a specific role in supporting children’s understanding of causal processes that extend over time, studied in the contexts of sinking, absorption, and solution analogical to physical, biological and chemical processes.

### 1.1 Spatial and spatial-temporal cognition

The varieties of spatial ability have been explored in considerable detail. Early studies found that it was not a unitary form of thinking; instead there were a number of spatial abilities, such as image generation, storage, retrieval, and transformation [10–13]. Recently, Newcombe and Shipley’s ([14]; see also [15]) much-cited theoretical model has distinguished between intrinsic/extrinsic and static/dynamic spatial abilities, as a basis for a classification of spatial tasks. Intrinsic skills involve the processing of spatial relations within objects without (static) or with alterations (dynamic) in these, as in mental rotation [15] and paper folding tasks [16]. Extrinsic skills address spatial relations between objects, again without or with transformations of these relations (e.g. spatial perspective taking [17]). Intrinsic abilities emerge around the age of four, whereas extrinsic dynamic abilities are detectible later in the development, becoming dissociated around the age of 9 to 10 years, at which point intrinsic spatial ability matures into an adult form [18]. However, in all of these the focus is on spatial configurations; even in dynamic tasks, there is no concern with the temporal dimension, merely with initial and final states.

The varieties of temporal ability have also been explored in considerable detail (see e.g. from [19–23]), with some underlining the fundamental role of temporal contiguity in cuing causal inference (see e.g. [24]). In this strand, research has elaborated that precedence, exact timing, and duration are important for causal perception/inference [25, 26], and there are key developmental achievements relating to this. For instance, there is (1) a shift from relying on temporal updating to being able to reason about time; and (2) the emergence of thought about other non-present time points (e.g. past, future). Various studies highlight that children below 5 years find it difficult to reason about time, in particular future situations [21, 27]. However, in all of these studies the focus is on temporal dimensions and there is no concern with spatial configurations, even if space and time meshed with each other in the task demonstrations (e.g. a moving body across space-time).

By comparison, only limited research has used tasks requiring a joint analysis of spatial changes that occur over time. Piaget [28] argued that primitive/early understanding of space and time was highly dependent on duration-distance judgements. For instance, he showed children two trains travelling on parallel tracks, and the majority said that the train that travelled the longer distance took the longer time regardless of relative rate of movement. He concluded that children did not reliably distinguish more complex spatial-temporal characteristics such as velocity until about age nine. Later, Piaget and Inhelder [29] proposed that children’s perception of space developed via gradual construction across three periods: ‘primitive perception of space’, ‘sensory-motor space’, and ‘representational space’. Regarding the emergence of temporal ability, they suggested that the apprehension of duration develops late and spatial cognition has priority in that it emerges first and provides the basis for temporal representations.

In contrast, Wilkening [30] found that preschool children demonstrated early implicit knowledge of time, speed, distance, and duration when the tasks involved more practical elements with which children had direct contact in their life. Similarly, working with pre-schoolers, Bullock et al. [31], Das Gupta and Bryant [32], and Gelman et al. [33] found children performed well on tasks where they were asked to place pictures in temporal order illustrating the spatial changes involved in a causal event such as a person cutting up an apple or dropping and breaking a wet cup.

As well as illustrating that children have good grasp of forms of spatial-temporal relationship from a young age, the latter studies signal a potentially important natural connection between the development of spatial-temporal and causal analysis. Further evidence in favour of both points is provided by research on intuitive physics. This has found that pre-school children perform competently on tasks requiring the capacity to predict phenomena which inherently involve spatial-temporal transformations, such as the trajectories followed by propelled objects (e.g. [34]; see [35], for a broader survey). However, none of this research has examined spatial-temporal analysis separately from causal analysis. This leaves it unclear whether–as has been argued to be the case for spatial cognition [36]–spatial-temporal analysis constitutes a distinct ability with its own developmental path, which children learn to deploy to support causal thinking. Investigating this possibility, and whether there is a typology of spatial-temporal cognition paralleling that of spatial cognition, are the primary goals of the present research.

### 1.2. Causal thinking and the role of spatial-temporal cognition

Research relevant to the role of spatial-temporal cognition in causal thinking has focused on two main strands: *causal mechanism*—which draws on inference as studied in the tradition of Hume [37] or Kant [38]; and *causal perception*–which emphasises perception in the tradition of Michotte [23].

In the first strand, mechanism refers to an inferred system of visible and invisible characteristics interacting systematically, whereby the same effects are consistently produced by the same causes [39]. A focus on mechanism is common across psychological views on causality that otherwise differ widely, from Bayesian accounts to force-dynamic accounts such as McCloskey’s [40] (see [26], [41], [42], also see [43], for demonstrations of mechanism reasoning with adults).

In terms of developmental research, Piaget [44] pioneered work on thinking about the unobservable mechanisms underpinning perceived regularities. However, his tasks asked children to explain either the nature of phenomena with which they had little direct contact, such as steam engines, or complex operational connections and transformations, such as the mechanism of bicycles. He concluded that young children were pre-causal, and the requisite skills emerge late. This has been refuted by demonstrations that pre-schoolers show concern for causal mechanism, if simple tasks with low verbal demands are used, and the operation of the mechanisms in question is familiar to them from everyday life or pre-test experience ([31], [45–48]).

Buchanan and Sobel [45], for instance, showed children that pressing one of two buttons made a light go on; the causal button had a sticker and was connected to the light by a wire or had a battery inside. Four-year-olds predicted that if the wire was switched, the other button would make the light come on; 3-year-olds, who did not yet understand wires, predicted this if the battery was switched. Neither age thought switching stickers would affect the outcome. This selectivity indicates concern with how causal influence was transmitted from cause to effect, beyond what was observed.

In work on causal perception, Michotte’s [24] pioneering studies on perceptual causality with adults documented that causal relations did not necessarily need to be inferred from regularities as Hume [37] suggested. Using the example of billiard balls, one causing another to move, he found that for the vast majority of participants when a cue ball (A) hit another ball (B), the motion of B was not perceived as its own, but instead was perceived as a simple continuation of A’s motion. Following Michotte’s paradigm, studies demonstrated that perceptual causality appears from infancy [49], [50]; undergoes development with different rates during childhood [51]; and matures into a form where perception of object interactions can be direct, immediate, without the assistance of prior experience, language, or causal learning. Even small manipulations of latencies, velocities, or direction of stimuli disrupt the perception of causality, indicating that this is stimulus-driven [52].

In both strands of work, however, causality is characteristically limited to distinct, clearly segmented events (A causes B to happen). This schema does not apply to continuous causal processes (e.g. the earth travelling around the sun), where there is no contiguity between distinct events. Natural phenomena that children encounter in school science commonly involve such temporally extended continuous processes, as when objects sink, dissolve or soak up water. However, primary age children appear to find making inferences about mechanism more difficult when they need to capture it from continuous processes, although those with better nonverbal ability do so more readily ([53, 54], see also S1 Appendix, and [55] for adult data). As explained further in the following sections, we hypothesize that spatial-temporal analysis provides a bridge between mere observation of continuous processes and their causal analysis, although causal analysis per se requires additional inference. This bridge comprises the use of three forms of spatial-temporal analysis: a) extracting key dimensions of information from states that change over time, effectively segmenting continuous processes into distinct steps (e.g. those involved in the transition from a sinking object being placed in water to it hitting bottom); b) conceiving of the sequence of dynamic transformations that underlie such observed change (e.g. recognising the rapid rate of sinking of a stone compared to a berry, as captured by the extent of spatial change per unit time when ordered into an overall sequence; cf. the sequencing demonstrated in [32]); and c) projecting these transformations onto past, present, and future experiences as the basis for anticipating and explaining the underlying causal mechanisms that produce them (e.g. by drawing inferences about the implied, but not observable imbalance between downward and upward forces) [53], [54], [56].

To test this hypothesis, a detailed research is required to elaborate on the development of spatial-temporal ability (i.e. the capacity to encode and mentally manipulate information about spatial changes over time) and whether this ability helps provide children with the basis for insights into natural mechanisms. As the present studies are the first to examine the role of spatial temporal analysis in continuous causal processes, we employed a range of tasks to assess whether these differentially predict children’s causal thinking about these. In this paradigm, spatial-temporal analysis is distinct from causal analysis. The tasks employed here, adapted from Piaget [28] and Wilkening [30], required *only* analysis of how spatial configurations changes over time, without any causal dimension being involved. In the ‘flow of liquid’ task children had to observe and recall segmented stages of how liquid flowed from an upper to a lower flask, marking on paper the levels in both flasks at each stage. The flask images were then scrambled, and children had to reconstruct the spatial configurations in the correct temporal order. They therefore needed to consider the sequences of dynamic changes in the flask system, principally when the liquid level in the top flask decreased, it increased in the bottom flask.

The other types of spatial-temporal task involved various objects (animals) moving at different speeds from various starting points towards a common goal. In the ‘speed’ task, children saw the beginning of the motion and had to extrapolate its end, i.e. a future spatial configuration. The ‘distance/time/velocity’ (DTV) tasks were similar, but provided less perceptual support, asking children to imagine in turn outcomes for one of three variables, the distance travelled, time taken or relative velocity of the objects, from information they were provided with on the other two variables. The tasks therefore employed different forms of spatial-temporal analysis: segmentation and ordering was captured by flow of liquid; perceptual extrapolation by speed; and visualisation of change by DTV. Each may be important ingredients–and predictors–of children’s reasoning about continuous causal processes, because they tap into the analytical and perceptual skills needed to structure these.

We also included variants of established measures of spatial thinking. There is growing interest in, and good evidence for, a link between spatial thinking and chemistry [57], physics [58], and success in science, technology, engineering, mathematics (STEM) fields [14], [59], [60]. However, the role of spatial ability in causal thinking has not received attention, and there is no existing analysis of the ways in which it might provide support. In order to close this gap in the literature we examine whether a) spatial-temporal ability is distinct from these, and b) spatial ability has any predictive value of its own as far as causal analysis of continuous processes is concerned. In doing so, we focused on intrinsic spatial skills, since causal analysis concerns change within systems. In a mental rotation task [61], children were asked to identify a target shape among foils in rotated positions. Here the spatial configuration within the system was static, and observers had to find the correspondence by mentally simulating its rigid motion. In the paper-folding task [62], children had to identify the configuration produced as a consequence of specific folds being made in a piece of paper. Although this task contains a dynamic aspect, the spatial characteristics of the paper are not intrinsically modified. In our view, therefore, both of these spatial tasks concern fixed systems, while the spatial-temporal tasks concern systems with spatial changes over time in the system itself, as captured by information about distance, duration, speed, and sequence of states, often described as vital cues for causal analysis.

### 1.3 Overview of the present research

The present research therefore explored the development of spatial-temporal ability and the relationship between this and spatial ability in predicting children’s thinking about continuous causal processes. It comprised two studies (N = 107, N = 124) with children aged 5 to 11 years.

In both studies, children were tested individually on the same causal tasks (sinking, absorption and dissolving), in which they saw two or more instances of each process that varied in speed. In the first study, they predicted what would happen before events were witnessed, as a measure of knowledge; described what happened, as a measure of observational skill; and explained their observations (e.g. why a stone sinks faster than a berry), as a measure of inference about operative causal variables and mechanisms connecting these to outcomes. In the second study, a more extended (five-stage rather than three) version of these tasks was employed in order to increase the number of observations on which children’s inferences were based.

Further tasks assessed spatial-temporal and spatial ability. In Study 1 (a) flow of liquid measured the capacity to reconstruct a sequence of segmented changes and the principle underlying these; and (b) speed examined perceptual extrapolation of relative speed of three objects. Similarly, two tasks assessed spatial ability: (a) mental rotation, and (b) paper folding. In the second study, two spatial-temporal tasks were used: flow of liquid remained, as its predictive power was high. Speed replaced by the distance-time-velocity tasks, measuring visualisation of change. Due to lack of predictive power of both previous spatial tasks, a different mental rotation task was also employed. In both studies, generic verbal and nonverbal ability were tested using WASI expressive vocabulary and block design. In order to test our hypothesis that spatial-temporal analysis provides a crucial bridge to causal analysis of continuous processes, the goal was to examine how children’s thinking about underlying causal mechanisms may link to their ability to analyse spatial-temporal structures above and beyond spatial and generic verbal and nonverbal abilities.

## 2. Study 1

Study 1 addressed two interrelated objectives. The first was to ascertain the developmental trajectory of spatial-temporal thinking in 5 to 11 year olds, in terms of sequence reconstruction within the flow of liquid task and extrapolation of perceived movement within the speed task

The second objective was to assess the predictive power of the spatial-temporal and spatial measures for children’s causal thinking regarding the three continuous processes (sinking, absorption, solution). Interest centred on the extent to which they predicted performance–especially in terms of identification of mechanisms–above and beyond verbal (expressive language) and nonverbal ability (block design).

### 2.1. Method

#### 2.1.1 Design

The study combined an individual differences and cross-sectional experimental design, employing three groups spanning the English primary school age range. Parents received a written consent form via schools explaining the target of the study, procedure, test materials, duration, and 299 the expected outcomes for the research. They were requested to provide their children’s demographics (age, language spoken at home, school year, parental occupation). Only children whose parents signed the consent form were included in the study. Children’s verbal approvals were also received before the start of each session. Children were not included in testing without their verbal consents even if their parental consent was available. This protocol approved by the UCL Institute of Education Research Ethics Committee, University College London.

Tasks were given to children in fixed order within a single one-to-one session: measures of verbal and nonverbal ability; three causal thinking tasks; two spatial-temporal tasks; and two spatial tasks. The causal, verbal and nonverbal tasks, and details of data relating directly to these (reported in [53] and described in S1 Appendix of the supporting information) are summarised briefly, as not being the main concern here.

#### 2.1.2 Participants

The sample consisted of 107 children, recruited with parental consent from three schools in London and Oxford, 35 from Year 1 (Y1), mean age = 6 years, 1 month, sd = 4.4 months; 33 from Year 3 (Y3), mean age = 8 years, 4 months, sd = 5.9 months; and 39 from Year 5 (Y5), mean age = 10 years, 3 months, sd = 5.9 months. Ethical approval was given by the authors’ institutional Research Ethics Committee. Responses to a parents’ questionnaire showed the sample included wide ethnic and linguistic variation (41.1% came from bilingual/trilingual homes), but was skewed towards the upper range in terms of socioeconomic background (72.9% of parents worked as professionals, and 93.5% had at least undergraduate degree qualifications).

#### 2.1.3 Materials and procedure

Testing took place in a quiet area within schools. Sessions lasted on average 37 minutes (min = 25, max = 55). Responses were recorded manually on score sheets, but children’s replies during the causal tasks were also audio-recorded for later scoring/checking.

*Causal tasks*. The causal tasks focused in turn on two contrasting instances of sinking (a stone and a blueberry sinking), absorption (a piece of tissue and blotting paper absorbing water), and solution (rock and table salt dissolving in water). The tasks were administered, scored and checked for reliability as described in S1 Appendix of the supporting information. For each pair of contrasts, children were asked to predict outcomes, as a measure of prior knowledge, before witnessing and describing what actually happened when the two instances were demonstrated alongside each other. They were then asked to explain the observed outcomes, as a measure of causal inference. Interest here centred on whether they could only identify basic factors (e.g. thinness of paper in absorption, grain size in solution), treat these as variables producing differential outcomes (e.g. relative thinness or grain size), or describe the mechanisms involved (e.g. object density, the role of water, holes in paper allowing water to rise, surface area affecting how rapidly water can penetrate). Data from these tasks were used to compute three types of measure:

1. Individual total scores for *prior knowledge*, *description*, and *explanation*. For prior knowledge, accurate prediction for each object was scored as 1. Therefore, the maximum score for each phenomenon was 2, and 6 across the three. Correct description similarly scored 1 per object, giving a maximum of 2 for each phenomenon, and 6 across them. The level of *explanation* was scored as 1 for identification of factors, 2 for variables, 3 for mechanisms. The maximum score across the three tasks was 9.
2. A *total causal score* combining the scores for each component (0–21; Cronbach’s α = .751).
3. The total number of *mechanism* level explanation responses made by children across the tasks (0–3).

*Measures of verbal and nonverbal ability*. The *expressive vocabulary* and *block design* subtests from the Wechsler Abbreviated Scale of Intelligence (WASI) (Wechsler, 2011) were administered and scored followed standard procedures, to provide measures of verbal and nonverbal ability.

**Fig 1:**
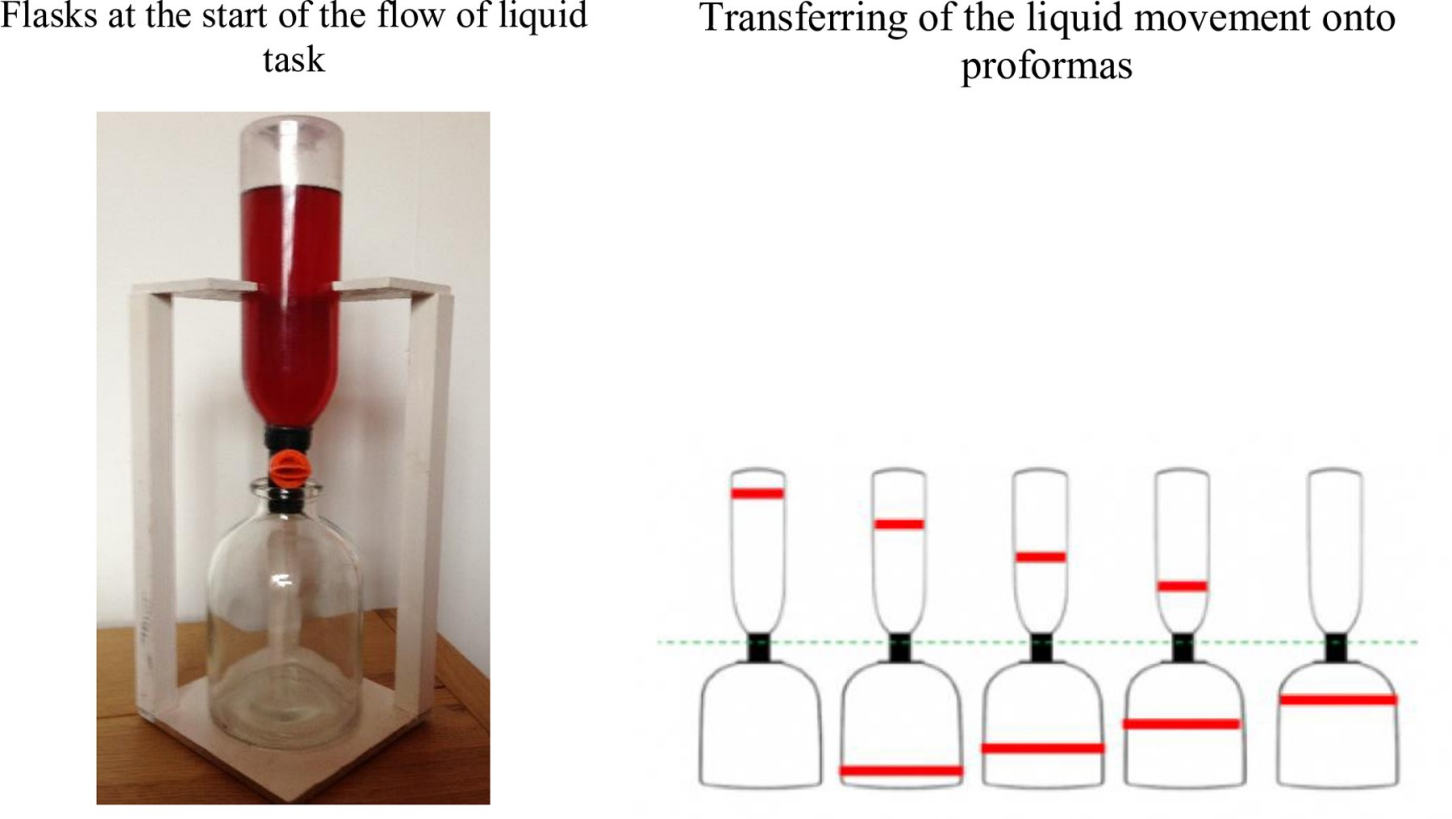
Flow of liquid task.

*Measures of spatial-temporal analysis*. The *flow of liquid* task, adapted from Piaget [28], examined children’s ability to analyze the flow of liquid from one container to another at successive time points, and reconstruct the sequence of change. It consisted of three stages. At the first, two flasks were presented one on the top of the other with a tap between (Fig 1). The upper flask (I) was filled with red-coloured water, while the lower (II) was empty. Children were given a proforma showing both flasks with a neck between them and they marked the respective levels in the flasks by drawing horizontal lines on the proforma. The liquid was then allowed to flow from I to II in four further steps, and the child marked the liquid level on a fresh proforma each time, being invited to correct any errors. At stage two, the proformas were shuffled and the child was asked to put them in order, again being invited to correct any errors. At the third stage, each proforma was cut in two, separating drawings of I from II, shuffled, and the child attempted to put them in order again. Scores were based on the number of drawings in the correct position at this stage, and could therefore range from 0–10.

**Fig 2:**
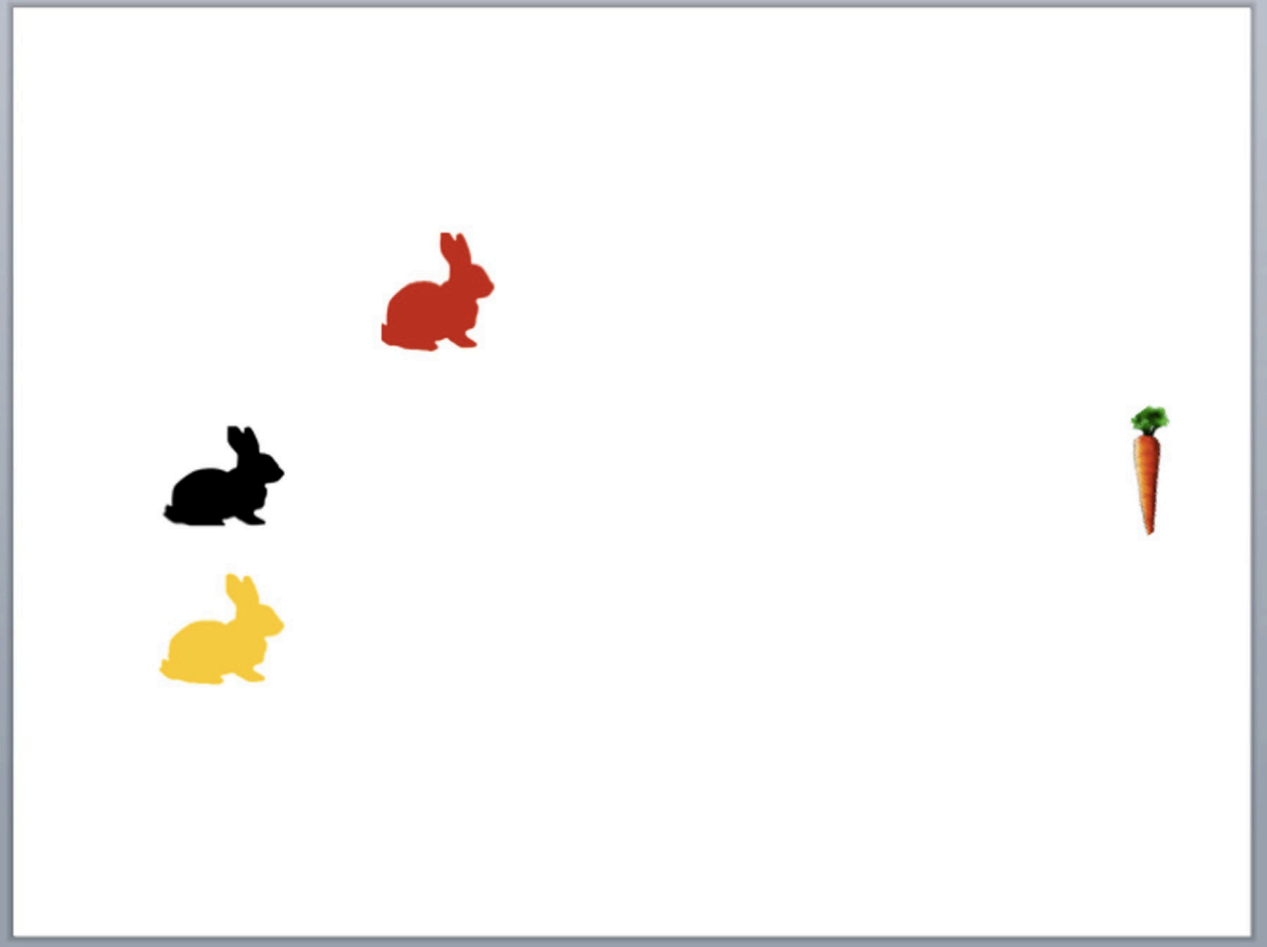
Example configuration of the bunnies at the start of a speed task trial.

In the *speed* task, children saw computer animations of three bunnies (red, yellow, black) racing towards a carrot from different start positions at different speeds (Fig 2), with the animation stopping before they reached it. Children judged which bunny would arrive at the carrot first. The task began with two practice items, followed by 17 trials gradually increasing in difficulty in order to capture the range of individual children’s ability from the most basic (i.e. where they are only able to accurately respond to very simple items) through to the most accomplished (i.e. where they can make accurate responses to most if not all items): the stop time reduced, from 6 to 4 seconds, as did the difference between the three bunnies in start point and relative speed, making differences in arrival time harder to distinguish, and the period available within which to track the differences shorter. The number of correct responses was recorded (0–17).

**Fig 3:**
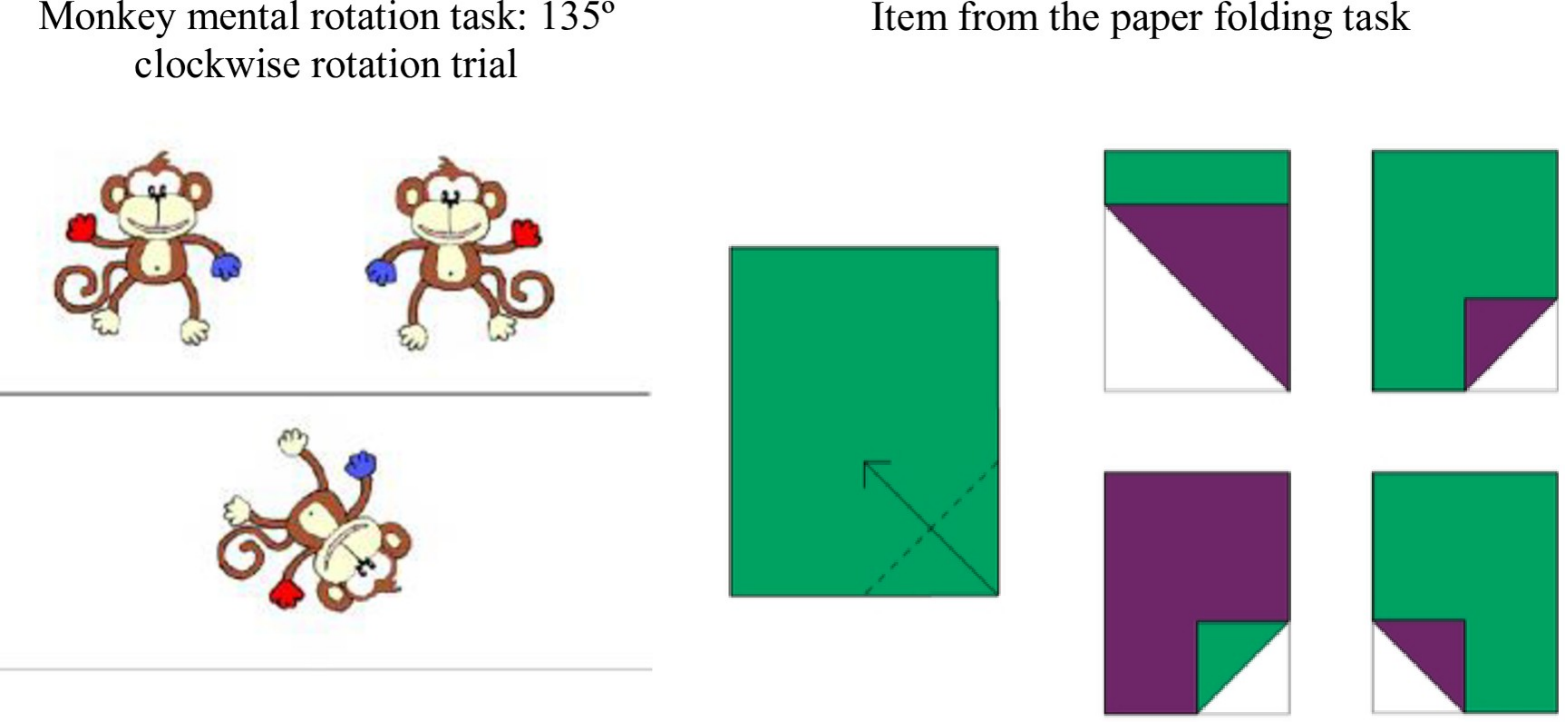
Spatial tasks.

*Measures of spatial analysis*. The *monkey mental rotation* task was based on the adaptation by Broadbent, Farran and Tolmie [63] from Shepard and Metzler [61]. Children saw laminated sheets with two mirror image cartoon monkeys above a horizontal line and one below at varying degrees of rotation from upright (Fig 3), and chose which of the two top monkeys matched the one underneath. Three practice trials were followed by 16 experimental trials (2 x 0° trials; 4 x 45° trials, 4 x 90° trials, 4 x 135° trials, with equal numbers of clockwise and anticlockwise rotations of each target monkey; and 2 x 180° trials), presented in pseudorandom order. The number of correct responses was recorded (0–16).

*Paper-folding* was adapted from Eliot and Smith’s [62] task by Harris, Newcombe and Hirsh-Pasek [16]. Children saw laminated coloured sheets of paper with markings indicating a fold, and selected from four options the one that represented the outcome (Fig 3). There were two practice items, and 14 trials, which increased in difficulty by employing more complex shapes and folds, and response options that were harder to distinguish from each other. The score was the number of correct responses (0–14).

#### 2.1.4 Statistical analysis

Mean scores by age group were computed for each of the indices of performance on the causal, spatial-temporal and spatial tasks, and the measures of verbal and nonverbal ability. In line with the first objective of the study, the developmental trajectory of performance on the two spatial-temporal tasks was examined in detail. One-way ANOVAs were also used to test for differences between age groups on these measures, and on each of the measures from the other tasks. Since there was significant skew on scores for spatial and generic ability, and concomitant non-homogeneity of variance, the Welch robust index was employed as the test statistic for these measures, as a conservative measure of the effects of age group [65]. Effect sizes are reported as partial eta squared (*η*_p_^2^). Main effects of age were followed up with planned tests assessing differences between each pair of age groups, using Bonferroni corrections to the criterion probability due to the multiple within-family comparisons.

In order to address the second objective of the study, patterns of relationship between the total causal task score and the measures of spatial-temporal and spatial ability were examined first of all using Pearson correlations, both raw and controlling for age and generic verbal and nonverbal ability. Estimates of the unique variance in causal task performance accounted for by each of these predictors were then obtained using hierarchical linear regression, with the order of entry of the non-generic measures guided by the relative strength of their correlation with the causal task score. Adjusted R^2^ is reported for the variance explained by the final model in this analysis, as less biased by sample size [64]. Potential confounds in these estimates of unique variance that might result from interactions or dependencies between predictors were checked for using the Hayes [65] methods for moderation and mediation analyses. The model of the combined effects of the predictors derived from the regression analyses was tested for fit to the data using path analysis. Finally, chi-square (χ^2^) analyses were used to examine the hypothesised relationship between category of spatial-temporal performance (perfect versus non-perfect scores for flow of liquid) and identification of mechanisms as opposed to variables or factors during explanation responses within the causal task.

The ANOVAs, raw correlations and chi-square analyses utilised data from all 107 participants who completed testing; data from one child were excluded for the partial correlations, regression and path analyses, due to missing date of birth information. Two-sided tests were used where relevant, with p < .05. The observed power for the regression analyses was 0.95, with α = .05, calculated using the post hoc routine within G-Power 3.1.9.2 [66].

### 2.2. Results

#### 2.2.1. Developmental trajectories

*Causal tasks*. The means for each age group on the causal task indices (see Table 1) illustrated that children performed well (relative to the maximum) on description, at a lower level on prior knowledge, and substantially less well on explanation, reflecting the low incidence of mechanism responses. One-way ANOVA nevertheless showed significant age-related progression on total causal score, F(2,104) = 24.250, p < .001, *η*_p_^2^ = .318, consistent with the effects on the constituent components noted in S1 Appendix of the supporting information; and on number of mechanism responses, F(2,104) = 5.182, p = .007, *η*_p_^2^ = .091. For the three main components, there were significant differences between Year 1 (Y1) and Y3, but not between Y3 and Y5; for mechanisms, there was a significant difference between Y1 and Y5, indicating later growth for responses at this level.

**Table 1:** Mean score (standard deviation) by age group on total causal score (max = 21), prior knowledge, description (max = 6), explanation (max = 9) and mechanism (max = 3).

	Y1	Y3	Y5
Causal total	10.63 (4.44)	14.42 (2.96)	15.97 (2.44)
Prior knowledge	3.26 (1.52)	4.15 (1.50)	4.85 (1.09)
Description	4.03 (1.60)	5.21 (0.99)	5.59 (0.68)
Explanation	3.34 (1.89)	5.06 (1.54)	5.54 (1.54)
Mechanism	0.03 (0.17)	0.30 (0.58)	0.44 (0.72)

**Fig 4:**
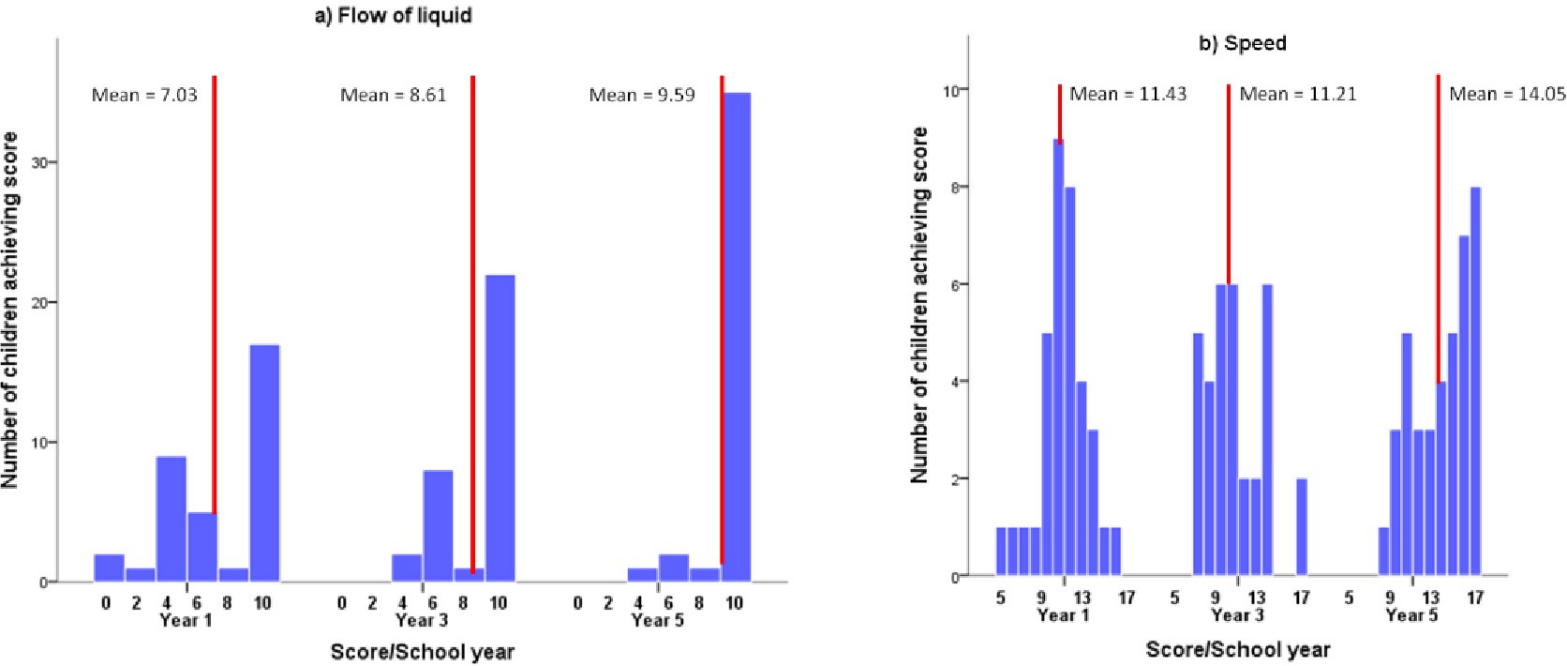
Profile of scores on a) flow of liquid (max = 10); and b) speed (max = 17).

*Spatial-temporal measures*. Response profiles for each age group on the flow of liquid and speed tasks (see Fig 4), and one-way ANOVAs showed age-related increases on both, but with growth occurring later for speed: for flow of liquid, F(2,104) = 11.180, p < .001, *η*_p_^2^ = .177, with significant differences between Y1 and Y3, but not between Y3 and Y5; and for speed, F(2,104) = 16.492, p < .001, *η*_p_^2^ = .241, with no difference between Y1 and Y3, but both significantly different from Y5.

For flow of liquid, there was steady growth with age in the percentage of children obtaining perfect scores of 10, with a diminishing tail of children who made one or more errors. In Y1, 17 children (48.6%) exhibited perfect performance, with scores otherwise ranging from 0 to 8 and more or less normally distributed across this range. There was a comparable pattern in Y3 and Y5, where 22 (66.7%) and 35 children (89.7%) respectively obtained perfect scores, and other scores fell between 4 and 8.

Looking at children’s performance across the three stages of the task, 20 (18.7%) initially failed to transfer liquid levels onto paper appropriately at stage 1, while 14 (13.1%) failed to put their drawings in sequential order at stage 2. These children also tended to make mistakes at stage 3: errors in drawing at stage 1 correlated at r = .24, p < .05, with errors in ordering at stage 2; and at .35, p < .001, with mistakes at stage 3; and similarly errors in ordering at stage 2 correlated at .20, p < .05, with mistakes at stage 3. Failure at stage 1 in particular therefore presaged failure at stage 3. Taken together, the data suggest a distinction between a growing number of children with age who had a perfect strategy for the task; and a declining number who failed to grasp it, and who made often multiple errors throughout.

Despite different item properties and later growth, performance on the speed task also suggested a dichotomy between children with a good grasp of task requirements, who increased in number with age, and children who made many errors. For Y1, scores showed a good fit to the normal curve. The mean for Y3 was virtually identical, but the distribution was more bimodal, as was that for Y5, despite a higher mean and a tendency towards ceiling for the upper group (see Fig 4).

*Spatial and control measures*. Table 2 shows the mean scores across age groups on the spatial and generic ability measures (vocabulary and block design). There was significant negative skew on scores for rotation and paper folding, and positive skew on scores for block design, due to the youngest group having a wider range and therefore a longer tail of low scores in the distribution of the negatively skewed variables, and the oldest having a wider range and longer tail of high scores on the positive. Vocabulary was normally distributed. One-way ANOVAs found significant increases with age on each: for rotation, Welch robust statistic = 13.901 (df = 2, 46.893); for paper folding, 47.983 (2, 60.628); for vocabulary, 54.093 (2, 67.790); for block design, 45.070 (2, 63.948); p < .001 for all. For rotation, there were significant differences between Y1 and Y5; for the other measures, all three age groups differed significantly. Progress on the spatial tasks was therefore slightly later than for flow of liquid, where there were no differences between Y3 and Y5; but earlier than for speed, where there were no differences between Y1 and Y3. However, performance on rotation nevertheless began to approach ceiling by Y3, in common with flow of liquid.

**Table 2:** Mean score (standard deviation) by age group on rotation (max = 16), paper folding (max = 14), vocabulary and block design.

	Y1	Y3	Y5
Rotation	12.89 (3.89)	14.15 (2.42)	15.64 (0.58)
Paper folding	5.03 (3.59)	7.97 (3.04)	11.28 (1.89)
Vocabulary	22.89 (5.29)	30.76 (5.86)	35.62 (5.20)
Block design	11.91 (6.08)	19.15 (9.52)	34.10 (13.25)

#### 2.2.2. What predicts children’s causal reasoning?

*Correlations between variables*. Zero-order Pearson correlations were computed to provide an initial assessment of the relationship between total causal score and all the potential predictors (see Table 3). All variables were positively correlated with each other. The relationship of the predictor variables to causal performance was linear, apart from scores on block design, for which the logarithmic relationship explained notably more variance, R^2^ for linear fit = .263; R^2^ for logarithmic fit = .368. Subsequent analyses used a logarithmic transformation of the block design score, log block design, in order to capture more accurately the variance explained by this measure of generic nonverbal ability.

**Table 3:** Zero-order and partial correlations between total causal score, verbal and nonverbal ability, spatial-temporal analysis and spatial ability (significant associations in bold).

	Causal total	WASI vocabulary	Block design (logarithmic)	Flow of liquid	Speed	Rotation	Paper folding
Causal total	1	.538***	.606***	.517***	.350***	.420***	.567***
WASI vocabulary	-	1	.679***	.437***	.419***	.382***	.619***
Block design (logarithmic)	-	-	1	.434***	.426***	.461***	.726***
Flow of liquid	.304**	-	-	1	.245*	.206*	.432***
Speed	.072	-	-	.020	1	.246*	.348***
Rotation	.173	-	-	-.021	.032	1	.501***
Paper folding	.144	-	-	.113	-.016	.235*	1

Zero-order correlations above diagonal, N = 107; partial correlations below diagonal, N = 106;

*p < .05,

**p < .01,

***p < .001.

When age in months, vocabulary and log block design were controlled for, only flow of liquid remained significantly associated with causal score. Speed was unrelated to any other variable, including flow of liquid. Rotation was related to paper-folding, and marginally to causal score, p = .081, due to the two being positively associated only in the youngest age group, for Y1, r = .231; for Y3, r = -.027; for Y5, r = -.069, controlling for vocabulary and log block design.

*Hierarchical regression models*. Hierarchical regression was used to examine the unique variance in causal performance accounted for by the different predictors. Taking total causal score as the dependent variable, age in months and the results of the WASI vocabulary test were entered in the first stage of the analysis. The other control variable, log block design score, was entered at the second stage, to assess its effects separately from those of vocabulary. The two spatial measures were entered in the third stage, to gauge their impact before the spatial-temporal measures were included. Speed was included on its own at the fourth stage, to check its predictive value before flow of liquid was entered at the fifth stage, since the latter appeared to be more strongly predictive. A term for the interaction between age and rotation was entered at the final stage, as the relation of rotation to causal performance appeared to be age-specific.

This analysis produced significant models and R^2^ change at each stage except the third and fourth (Table 4). Age and vocabulary were significant predictors at the first stage, but both dropped out in favour of log block design when that was included. The inclusion of the spatial measures had little impact other than to reduce the beta for log block design. The inclusion of speed had no appreciable effect either. However, flow of liquid was a significant predictor when entered at the fifth stage, explaining a further 5.1% of the variance. The interaction between age and rotation score became a further significant predictor at the sixth stage alongside flow of liquid and log block design, accounting for an additional 3.7% of variance. We also explored the outcomes for other sequences of entering the predictors. When the spatial measures were added to the model before log block design, they reduced the beta for vocabulary score, indicating that they shared variance with generic verbal as well as nonverbal ability. When the spatial-temporal measures were entered before the spatial and nonverbal, flow of liquid now explained an additional 7.4% variance, suggesting that it shared variance with those measures.

**Table 4:** Hierarchical regression analysis with total causal score as dependent variable (significant predictors in bold).

Model	M1	M2	M3	M4	M5	M6
Predictor	β					
Age in months	.332**	.207	.154	.146	.110	.155
WASI vocabulary	.310**	.135	.111	.100	.060	.040
Block design (log)		.383**	.279*	.266*	.231	.249*
Rotation			.128	.125	.139	-.085
Paper-folding			.131	.133	.089	.109
Speed				.060	.054	.097
Flow of liquid					.261**	.266**
Age x rotation						-.278**

AdjR^2^ = .494; *ΔR*^*2*^ = .347*** for M1; .071** for M2; .024 for M3; .003 for M4; .051**for M5; .037** for M6.

*p < .05.

**p < .01.

***p < .001.

These models were also run for the three causal components, prior knowledge, description and explanation, and produced similar outcomes, except that flow of liquid was the only predictor of all components, including explanation. The effects of generic nonverbal ability were fully superseded by flow of liquid, except for prior knowledge. The influence of the spatial measures was restricted to description (see S2 Appendix of the supporting information).

In sum, the regression model for total causal score had flow of liquid, log block design and the interaction between age and rotation as significant predictors. Since the beta for the interaction term was negative (i.e. the effect reduced as age increased), this indicates that rotation had a greater impact on causal performance for younger children, in line with the effects seen in the partial correlations. The impact of spatial ability was restricted to this. Similarly, the logarithmic relationship of block scores to total causal scores indicates a steeper slope for the relationship between generic nonverbal ability and causal performance at lower levels, suggesting that this is where nonverbal ability was most discriminating. The linear effect of flow of liquid performance indicates, in contrast, that this measure of spatial-temporal analysis distinguished lower from higher levels of causal performance. This ability appeared to be largely independent not just of nonverbal and spatial ability but of verbal ability too. Moderation and mediation analyses, using the Hayes [65] method, confirmed the lack of interaction between flow of liquid and vocabulary in the prediction of causal scores; and that the relationship between flow of liquid and causal performance was primarily a direct one, not mediated by vocabulary.

**Fig 5:**
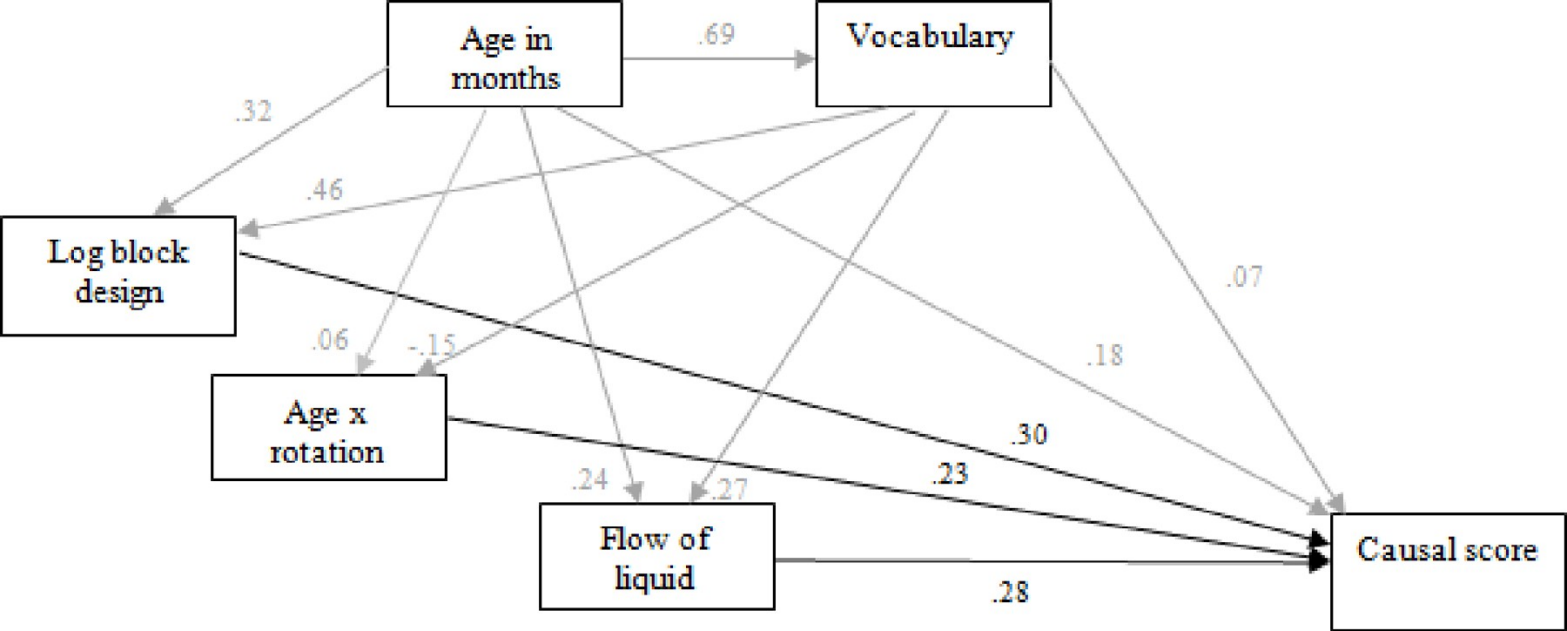
Path model including standardised coefficients for the effects of flow of liquid, log block design, age × rotation, age and vocabulary on causal score (subsidiary relationships in grey).

*Path analysis*. Path analysis using a maximum likelihood approach was employed to test the overall fit of the regression model for total causal score, treating age and vocabulary as background influences on the predictors (Fig 5). The path coefficients showed the direct effects of flow of liquid and log block design were near-equal in strength, and substantially stronger than the direct effects of age and vocabulary. The interaction between age and rotation also had a stronger effect than these. Both age and vocabulary had a stronger influence on log block design than on flow of liquid. This model provided a good fit to the data overall, χ^2^ = 3.526, df = 3, p = .317.

#### 2.2.3. Identification of causal mechanisms

The regression and path analyses illustrated that flow of liquid predicted causal scores, including explanation, better than either of the spatial tasks. Follow up chi-square analyses were used to examine more specifically how perfect reconstruction of the spatial-temporal states of the flasks linked to higher levels of causal explanation (Table 5). As can be seen, on each of the three causal tasks, the percentage of children exhibiting perfect scores was highest among those who identified the relevant mechanism (i.e. who had explanation scores of 3). The chi-square results for explanation score by perfect versus non-perfect flow of liquid score were significant for absorption, χ^2^ = 16.549, p = .001, and solution, χ^2^ = 9.102, p = .028, though not for sinking, χ^2^ = 4.154, p = .245; df = 3 for all. Taken overall, of those who mentioned mechanism across any of the three causal tasks, 85.7% had a perfect flow of liquid score; of those who never mentioned mechanism, it was 65.1%.

**Table 5:** Percentages of children obtaining perfect scores on the flow of liquid task at each level of explanation response.

	Explanation score			
Causal task	3 (mechanism) %	2 (variable) %	1 (factor) %	0%
Sinking	77.8	72.5	60.0	46.0
Absorption	86.7	76.6	63.6	29.4
Solution	100.0	79.2	69.2	53.7

These data illustrate at a high level of resolution our hypothesised link between spatial-temporal analysis and inference of mechanism.

### 2.3. Discussion

Study 1 had two key findings. First, children’s ability to analyse spatial-temporal transformations improved with age, both their ability to reconstruct a sequence of spatial states, and their ability to extrapolate future positions. However, the latter exhibited later development, and therefore appeared to be more difficult. Second, the ability to reconstruct a spatial-temporal sequence was a crucial predictor of children’s causal performance as a whole; this was true also when we ran the same analyses with prediction, description and explanation elements separately. Despite exhibiting similar developmental trends to spatial-temporal ability, spatial ability in the form of mental rotation only predicted the causal performance of younger children.

However, although there was clear and consistent evidence that flow of liquid predicted children’s causal performance very well–their inference of mechanism especially–the other spatial-temporal measure, speed, did very little. It is also possible that the limited number of observations made for each process constrained children’s ability to identify mechanisms, potentially suppressing the predictive power of the spatial measures. We therefore do not strictly know from these results whether a) spatial-temporal analysis in general is important to children’s reasoning about natural causal processes, or whether what is predictive is some more specific property of the flow of liquid task; or b) if greater exposure to instances of each process would change the picture. Study 2 therefore extended the methodology; employed a more diverse sample for greater generality; and involved further spatial and spatial-temporal measures.

## 3. Study 2

Study 2 addressed the same research questions as Study 1. In terms of the methodology, the causal tasks required more observations, and corresponded to a more realistic scientific procedure, with the focus again on contrasting speed of effect within sinking, absorption and solution. The scientific procedure followed this sequence: observation, description, prediction, justification, testing, explanation. The observation and description stages were as before, but there was no initial prediction from prior knowledge. Instead, prediction for three further objects followed initial observations, so children’s predictions could be informed by these demonstrations as well as prior knowledge. Children were also asked to justify why they thought their predictions were correct. They then tested their predictions and finally gave explanations of what they had observed. Children thus had a more active role in the procedure, and opportunity to think about each process across five rather than two examples before drawing concluding causal inferences. This procedure aimed to elaborate whether increased exposure promoted better causal performance. Pilot testing indicated that children gave more extensive explanations for these five-item comparisons, which allowed responses that simply identified causal variables to be differentiated from those that addressed the *joint* operation of these (albeit without mention of mechanism), indicating greater coordination of the information available from observation. The scoring system for explanation therefore captured this difference and examined whether it constituted a further interim step in children’s progress towards awareness of mechanism.

On the spatial-temporal measures, the flow of liquid task was retained, given its importance in Study 1. However, the water level changed in six steps rather than five, to enhance its discriminating power among older children who were typically at ceiling on the five-step version. The speed task was solved mainly by perceptual prolongation of the trajectories, similar to anticipating the throw of a ball, rather than thoughtful prediction. It was replaced by a set of tasks requiring judgements of one of distance, time or velocity (DTV) from information about the other two of these dimensions, based on Wilkening [30]. The DTV instead required mental simulation of movement against a time count. This aimed to elaborate whether it was spatial-temporal analysis involving mental construction of a sequence of change that was predictive of causal performance.

Since the monkey rotation task failed to discriminate older children’s performance especially, it was replaced by Stefanatos, Buchholz, and Miller’s [67] task adapted from Shepard and Metzler [61], which demonstrated good discrimination across the primary age range performance. It also used physical materials, providing a better parallel to the materials used in the causal reasoning and flow of liquid tasks. Given the overlap of paper-folding with rotation in Study 1, and its limited predictive power, this was dropped.

### 3.1 Method

#### 3.1.1. Participants

Participants were 124 children from three primary schools in Oxford (36 from Y1, mean age = 5 years, 11 months, sd = 3.8 months; 45 from Y3, mean age = 7 years, 11 months, sd = 3.6 months; 43 from Y5, mean age = 9 years, 9 months, sd = 5.1 months). Of these, 65 (52.4%) had a monolingual (English) home environment, and 59 (47.6%) came from bilingual/multilingual homes, exhibiting similar variation to Study 1. The socioeconomic background was more broadly representative, with 20 (16.1%) of parents being unemployed, 32 (25.8%) manual workers, 48 (38.7%) self-employed/non-manual workers, and 24 (19.4%) professionals. In total, 35 parents (28.2%) had only GCSE qualifications; 21 (16.9%) had A levels; 38 (30.6%) had an undergraduate degree; 22 (17.7%) had a postgraduate/professional qualification; and 8 (6.5%) had doctoral degrees.

#### 3.1.2. Design, materials and procedure

Design, age groups and task order were as in Study 1. Verbal and nonverbal ability were assessed as before. Test sessions (average length 41 minutes, min = 28, max = 56,) took place as in Study 1, except for the changes noted below.

**Fig 6:**
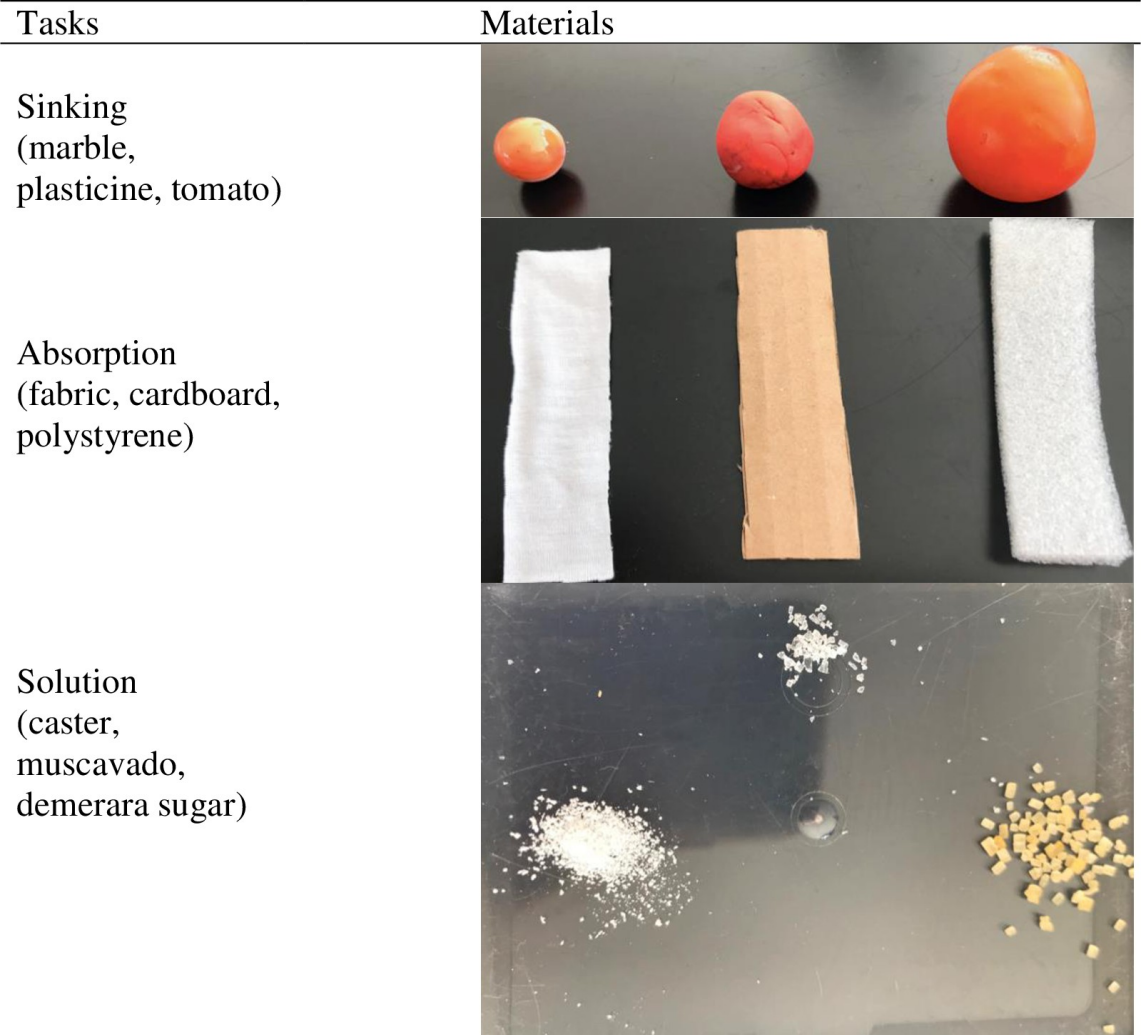
Materials in the correct order of prediction for the causal reasoning tasks.

*Causal tasks*. Each causal task had five stages: children (1) inspected the two contrasting materials from Study 1 (except a grape replaced the blueberry), watched them sink/absorb/dissolve in water, and *described* what they saw; (2) inspected three further contrasting materials (Fig 6) that sank/absorbed/dissolved at different rates (see S3 Appendix of the supporting information for details), and *predicted* which would sink/allow water to rise/disappear fastest, next fastest and slowest; (3) *justified* why they thought their predicted order was correct; (4) tested their predictions with the aid of the experimenter, to evaluate whether they were correct, and finally (5) *explained* why they thought things had happened in the way they had seen across the various observations, including the initial ones, of which they were reminded (inference).

Scores for children’s description, prediction, justification, and explanation responses were arrived at in the same way as Study 1, using the modified system shown in S4 Appendix of the supporting information (example explanation responses illustrating the additional level of scoring for coordination of causal variables are also shown). Composite measures were computed for each response component (0–3 for *description*, 0–9 for *prediction* and *justification*, 0–12 for *explanation*), and for *total causal score* (0–33; Cronbach’s α = .724). As in Study 1, we also noted the number of *mechanism* level responses made by children (0–3).

*Measures of spatial-temporal analysis*. The *flow of liquid* task was identical to Study 1, except that the liquid flowed from upper to lower flask in six rather than five steps, giving children six drawings to rearrange at stage two, and 12 at stage three. Scores could therefore range from 0–12.

Children completed three related *distance/time/velocity (DTV)* tasks [30], displayed in Powerpoint, which required them to makes estimates of each of distance, time and velocity in that order, by integrating information about the other two dimensions.

**Fig 7:**
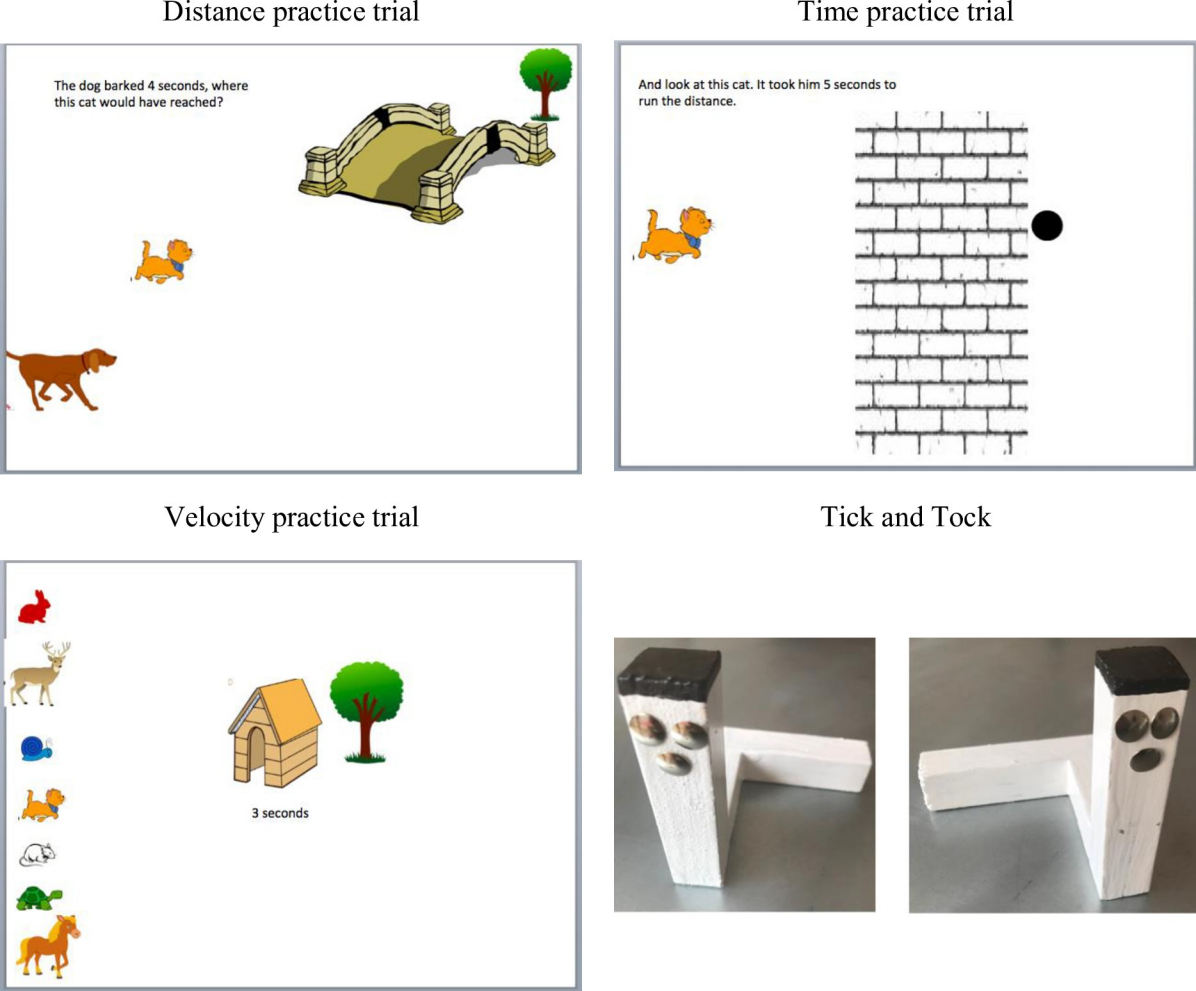
Materials for the distance/time/velocity and rotation tasks.

For *distance judgements*, children saw a cartoon cat, mouse, and turtle, and were told these animals differed in speed (cat fastest, turtle slowest, mouse in-between). They then imagined how far each would run in a fixed time, counted out loud by the experimenter, to escape from a barking dog. There was one practice trial of 4 seconds involving just the cat (Fig 7). During this, the experimenter and the child agreed on a calibration (after 4 seconds cat would be in the middle of the bridge). This practice trial was followed by three test items: all three animals appeared in the same starting positions relative to dog and bridge, with the dog barking for 6, 4 and 2 seconds. Children pointed to where each animal would have run each time. They received 2 points per item if their answer was fully correct (based on extrapolation from the initial illustration, relative animal speed and time), 1 for partially correct answers (correct distance for some but not all three animals), and 0 for completely incorrect answers. Scores could therefore range from 0–6.

For *time judgements*, children saw only one animal (either cat, bunny, or turtle) and estimated, by counting out loud themselves, how long this animal would take to run to a fixed point. The first part of the run was animated, with the second half concealed behind a wall. There were two practice trials, bunny and cat (Fig 7), during which children were helped to count appropriately for calibration, followed by three test items in the order turtle, cat, bunny. The cat and bunny in the test items differed in speed from the practice items. Children received 2 points per item if they counted to the correct time intervals (e.g. it took cat sometime between 4 and 6 seconds to reach to the point), 1 if they counted to one second either side (e.g. it took cat 3 or 7 seconds to reach to the point), and otherwise 0. Scores could therefore range from 0–6.

For *velocity*, children had to judge which of seven animals (deer, horse, cat, bunny, mouse, turtle, snail) were fast enough to make it to a house at varying distances in a given period of time, counted out loud by the experimenter. There was one practice trial (Fig 7) in which the animals had 3 seconds to run, and children were helped to the conclusion that just the horse, deer and cat would get to the house mid-screen while ensuring that any judgement of relative speed came from the child themselves. They then saw three test items, with a) house mid-screen, 4 seconds, b) house towards the close side, 2 seconds, and c) house towards the far side, 6 seconds. For each item, children were given 2 points if they identified a predetermined set of ‘winners’ (calibrated against the practice trial), 1 if they missed some out or included others, and otherwise 0. Scores therefore ranged from 0–6.

A total DTV score (0–18) was also computed across the three tasks, partial correlations controlling for age, r_distance/time_ = .417, r_distance/velocity_ = .363, r_time/velocity_ = .180, p < .05 for all; 56.6% shared variance in the first factor of a principal components analysis, with loadings of .840, .725, .684 for distance, time and velocity respectively.

*Measure of spatial ability*. In the *mental rotation task*, children saw two wooden stick figures, Tick and Tock [67], and were instructed that they would see photos (colour, pasted on to laminated A5 cards), sometimes of Tick and sometimes of Tock, and had to say which it was. There were three practice trials, during which any errors were corrected. These were followed by 16 test trials (2 x 0°, 4 x 45°, 4 x 90°, 4 x 135°, 2 x 180°, balanced across target and clockwise/anticlockwise rotations), presented in pseudo-random order. The score was the number of correct answers (0–16).

#### 3.1.3 Statistical analysis

The approach adopted to statistical analysis was identical to that employed for Study 1 in terms of both the hypotheses tested and the techniques employed to do so. Analyses utilised data from all 124 participants.

### 3.2 Results

#### 3.2.1. Developmental trajectories

*Causal tasks*. Table 6 shows the means for each age group on the causal task indices. Relative to the scales employed, performance was highest on description and prediction, as in Study 1. Performance on explanation lagged behind, and to a greater extent than in Study 1. Similarly, only 15.3% of children exhibited mechanism level responses on one or more tasks here, against 19.6% previously. The largest percentage of explanation responses for all three causal processes focused on relevant variables (scores of 2), though responses that explicitly coordinated these (scores of 3) were relatively common– 53% vs. 18.3% on average across the three tasks. Justification responses were notably poorer, with the majority restricted simply to identification of a single key variable (72.6% on average), probably reflecting the narrow pragmatic demands of the request to account for predictions.

**Table 6:** Mean score (standard deviation) by age group on total causal score (max = 33), description (max = 3), prediction, justification (max = 9), explanation (max = 12) and mechanism (max = 3).

	Y1	Y3	Y5
Causal total	15.80 (5.21)	16.77 (5.12)	19.27 (3.23)
Description	2.22 (0.83)	2.53 (0.59)	2.74 (0.44)
Prediction	5.97 (1.93)	5.69 (1.68)	6.42 (1.64)
Justification	2.58 (1.25)	2.87 (1.29)	3.49 (1.37)
Explanation	5.03 (2.36)	5.69 (2.56)	6.63 (1.75)
Mechanism	0.06 (0.23)	0.16 (0.42)	0.28 (0.50)

One-way ANOVAs were used to test age-related progression, which was significant for total causal scores, F(2,121) = 6.192, p = .003, *η*_p_^2^ = .093, and each component except prediction: for description, F(2,121) = 6.811, p = .002, *η*_p_^2^ = .101; for justification, F(2,121) = 5.065, p = .008, *η*_p_^2^ = .077; for explanation, F(2,121) = 5.096, p = .008, *η*_p_^2^ = .078. In each case, there were significant differences between Y1 and Y5, with Y3 intermediate. There was a marginally significant effect of age on mechanism level responses, F(2,121) = 2.936, p = .057, *η*_p_^2^ = .046.

In general, effects were smaller than in Study 1, and scores were lower, comparatively, tending to cluster around the mid-range of the scale: other than for description, they were normally distributed. Growth also took place later than in Study 1, suggesting either the tasks were more taxing, the sample had lower ability, or both. The lack of age effect for prediction does not appear to be attributable to children reaching ceiling early, but to performance commonly being only partially accurate.

**Fig 8:**
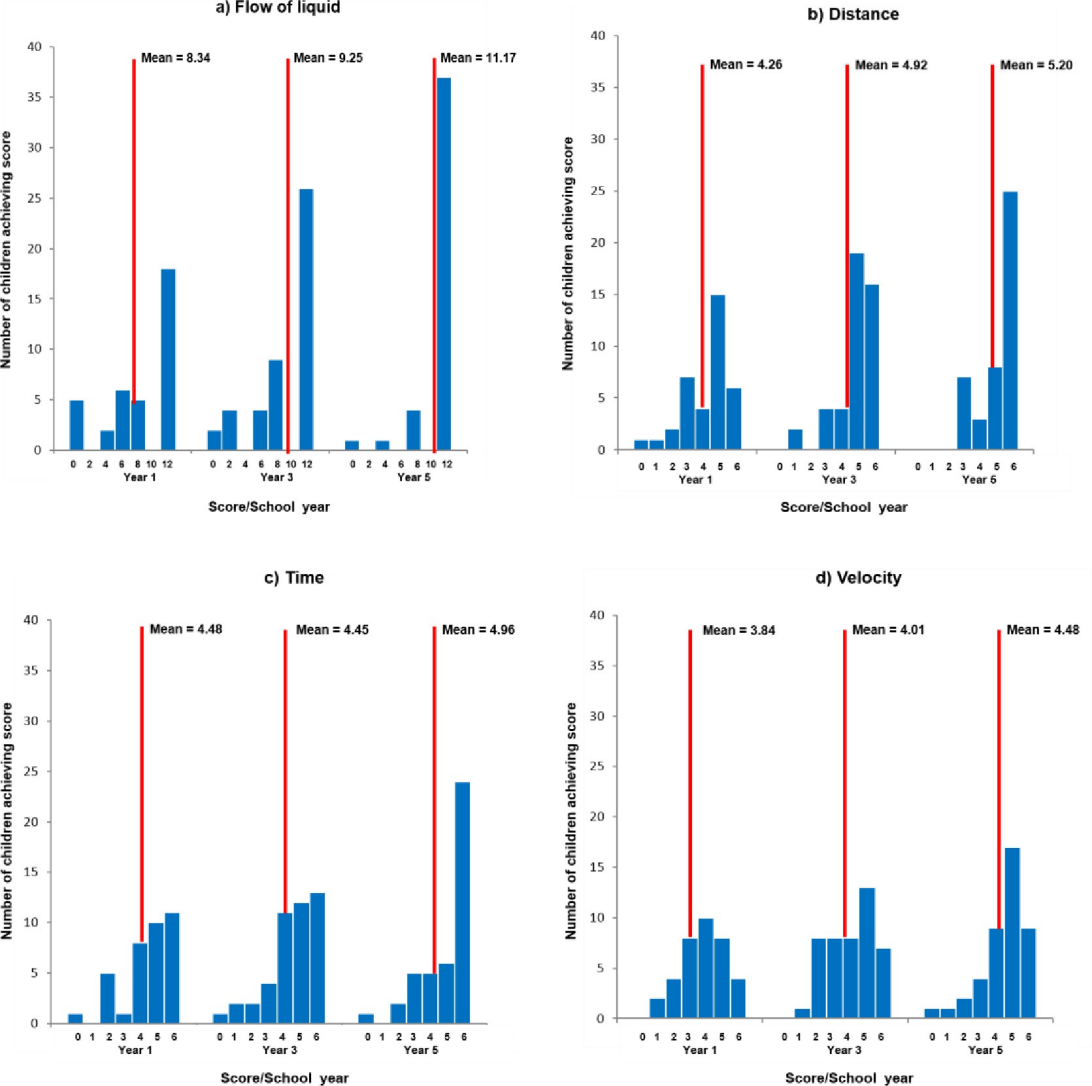
Profile of scores on a) flow of liquid (max = 12); b) distance; c) time; and d) velocity (max = 6 for all).

*Spatial-temporal measures*. Age-related response profiles for flow of liquid and DTV are shown in Fig 8. For flow of liquid, the pattern of scores was similar to that found in Study 1: the number of children obtaining perfect scores of 12 increased with age– 18 children (50%) in Y1; 26 (57.8%) in Y3; and 37 (86%) in Y5 –and there was a diminishing tail of those who made one or more errors. Thus the six-step version was not obviously harder. One-way ANOVA showed age-related progression, F(2,121) = 6.625, p = .002, *η*_p_^2^ = .099, with Y1 and Y3 equivalent, but lower than Y5, in line with the later development seen on the causal performance indices.

Although fewer children achieved maximum scores, performance on the DTV tasks was otherwise similar to flow of liquid. There was the same increase with age in high scores and reducing tail of those making more errors. However, this was most obviously the case for distance; time and more particularly velocity showed broader distributions of scores, suggesting they may have been slightly more demanding. One-way ANOVAs found significant increase with age on distance, F(2,121) = 5.423, p = .006, *η*_p_^2^ = .082, with Y1 significantly different from Y5, and Y3 intermediate; but not on time or velocity, although both showed upward trends with age. One-way ANOVA confirmed a significant effect of age on the total DTV score, F(2,121) = 4.117, p = .019, *η*_p_^2^ = .064, with Y1, mean = 12.58, significantly different from Y5, 14.56; and Y3, 13.33, intermediate.

*Spatial and control measures*. Table 7 shows mean scores by age group on the spatial and general ability measures. There was significant negative skew on rotation and vocabulary, and positive on block design. One-way ANOVAs by school year found significant increases with age on each: for rotation, Welch robust statistic = 15.004 (df = 2, 73.799); for vocabulary, 51.303 (2, 77.705); for block design, 24.229 (2, 79.791); p < .001 for all. For rotation, Y1 and Y3 were equivalent, but significantly lower than Y5, the later growth suggesting that in this sample the Tick and Tock task was more discriminating in the higher primary age range than the rotation task of Study 1, with performance broadly comparable to that on the flow of liquid and DTV tasks.

**Table 7:** Mean score (standard deviation) by age group on rotation (max = 16), vocabulary and block design.

	Y1	Y3	Y5
Rotation	12.43 (2.88)	12.99 (2.12)	14.85 (1.74)
Vocabulary	22.48 (5.39)	29.05 (5.09)	34.01 (4.56)
Block design	12.43 (5.63)	16.45 (7.12)	24.01 (8.97)

#### 3.2.2. What predicts children’s causal reasoning?

*Correlations between variables*. As in Study 1, correlations were used to provide an initial assessment of the relationship between the total causal score and the predictor variables. Flow of liquid tended toward an exponential rather than linear relationship with the causal indices: for total causal score, R^2^ for linear fit = .342 [F(1,122) = 63.546, p = .000], for exponential fit = .357 [F(1,122) = 67.810, p = .000]. Although this difference was not statistically significant, it nevertheless suggests that flow of liquid was marginally more discriminating of causal performance in the upper range of scores, reflecting the different causal task and sample characteristics. We therefore used an exponential transformation of flow of liquid scores in subsequent analyses, in order to capture more accurately the variance explained by this measure. The relationships for block design were logarithmic as in Study 1, and the logarithmic transformation of this measure was therefore used for the purposes of analysis, as before.

The total causal score showed positive associations with all the predictors (Table 8). The main predictors were also all positively correlated with each other. In this sample, level of parental education–but not parental occupation–was also weakly correlated with causal performance, though it was unrelated to any of the other predictors.

**Table 8:** Zero-order and partial correlations between measures (significant associations in bold).

	Causal total	WASI vocabulary	Block design (logarithmic)	Flow of liquid (exponential)	DTV total	Rotation	Parental education
Causal total	1	.531***	.480***	.600***	.440***	.337***	.191*
WASI vocabulary	-	1	.530***	.486***	.387***	.431***	.013
Block design (logarithmic)	-	-	1	.497***	.421***	.458**	.071
Flow of liquid (exponential)	.410***	-	-	1	.541***	.347***	.127
DTV total	.230*	-	-	.381***	1	.442***	.056
Rotation	.096	-	-	.093	.285**	1	-.007
Parental education	.176			.115	.023	-.016	1

Zero-order correlations above diagonal, partial correlations below; N = 124;

*p < .05,

**p < .01,

***p < .001.

Controlling for age in months, vocabulary and log block design, the only significant predictors of causal score were flow of liquid, as in Study 1, and now DTV, the other spatial-temporal measure. Parental education was marginal, p = .053. Flow of liquid and DTV were related to each other, while DTV also correlated with rotation. Unlike Study 1, there was no sign that rotation had an age-related effect.

*Hierarchical regression models*. Hierarchical regression examined the unique variance accounted for by the predictors with total causal score as dependent variable. Predictors were entered in the equivalent order to the Study 1 analyses, except that no age x rotation interaction term was included.

The analysis (Table 9) produced significant R^2^ change at every stage except the third, when rotation was entered. Age was a negative predictor throughout, becoming significant from the second stage on; this appears to be a function of its relationship to residual variance not explained by other predictors, since it was positively associated with causal scores (r = .285, p = .001). In contrast to Study 1, vocabulary was a strong positive predictor, though its beta dropped at each successive stage, especially when log block design and flow of liquid were included, suggesting that in this more diverse sample, it was related to both causal performance and spatial-temporal analysis, as well as to nonverbal ability. Block design was a significant predictor from the second stage onward, though its beta dropped with the inclusion of DTV and flow of liquid, again indicating a relationship with spatial-temporal analysis.

**Table 9:** Hierarchical regression analysis with total causal score as dependent variable (significant predictors in bold).

Model	M1	M2	M3	M4	M5
Predictor	β				
Age in months	-.136	-.245*	-.260*	-.229*	-.224*
WASI vocabulary	.624***	.519***	.503***	.457***	.370***
Block design (log)		.336***	.311**	.262**	.178*
Rotation			.090	.031	.037
DTV total				.199*	.068
Flow of liquid (exp)					.363***

AdjR^2^ = .452; *ΔR*^*2*^ = .292*** for M1; .075*** for M2; .006 for M3; .028* for M4; .077*** for M5. *p < .05.

**p < .01.

***p < .001.

The analysis confirmed that the new spatial-temporal measure, DTV, explained unique variance, independent of vocabulary, spatial, and nonverbal ability (Model 4). Its beta dropped sharply when flow of liquid was added, however, confirming its overlap in variance with the other spatial-temporal index. Flow of liquid was the stronger predictor of causal performance, explaining a further 7.7% of variance when it was included, more than in Study 1, with a larger final beta. Taken together, the two spatial-temporal measures explained 10.5% of variance. Rotation had no predictive value, though its entry reduced the beta for vocabulary and log block design, confirming it overlapped with both, as in Study 1.

The same models were also used to examine explained variance in each of the component scores, and produced similar results: DTV and flow of liquid remained clear but overlapping predictors of all components, especially explanation; vocabulary was generally strong; and log block design was weaker (see S2 Appendix in the supporting information). The models were also rerun with parental education at the final stage, to check whether it added predictive power. It was a significant predictor only for justification, with a beta of .158, p = .036.

**Fig 9:**
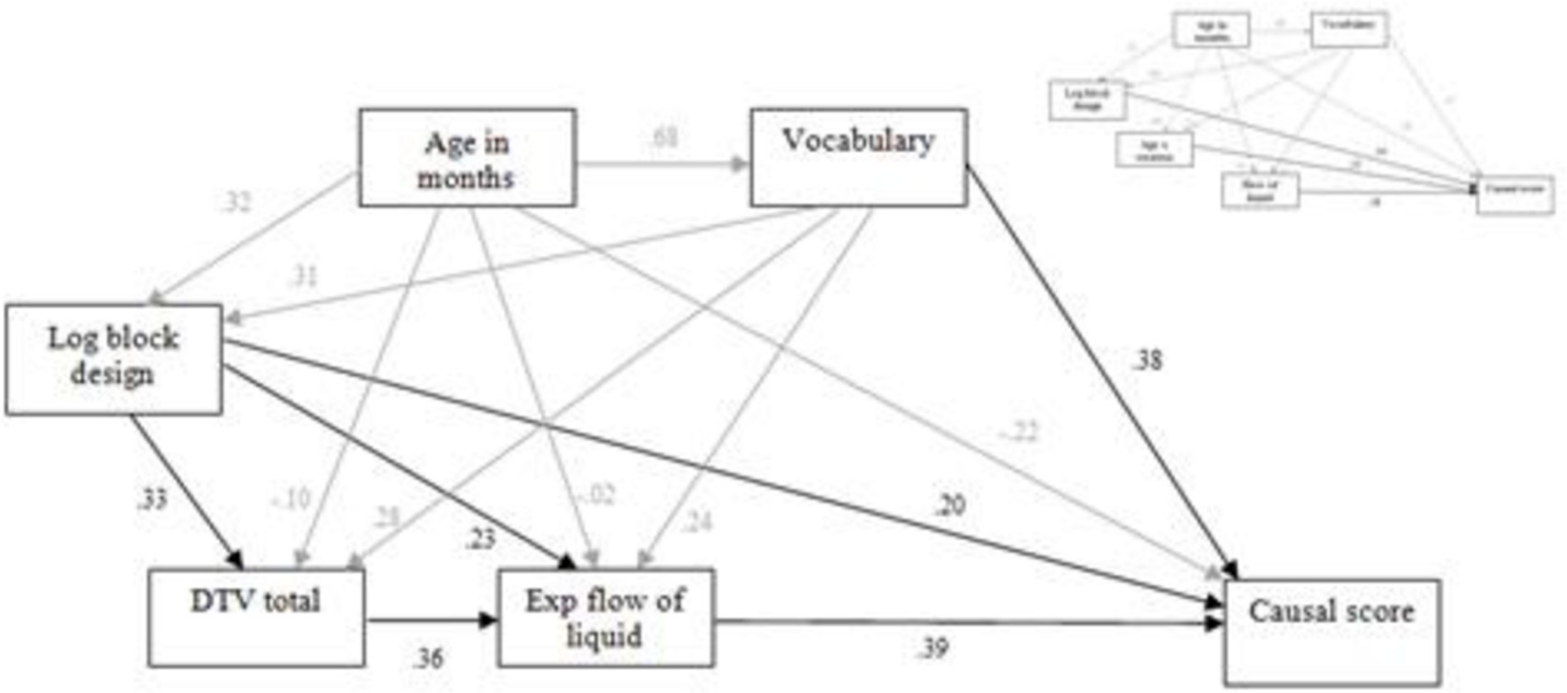
Path model including standardised coefficients for the effects of exp flow of liquid, DTV, log block design, age and vocabulary on causal score, with (top right) the model for Study 1 shown for comparison (subsidiary relationships in grey).

*Path analysis*. The overall fit of the model indicated by the regression analysis was examined using path analysis. The hierarchical output suggested that flow of liquid and vocabulary, and to a lesser extent block design, were direct predictors of causal performance in this sample. The pattern of reduction in beta values also indicated that block design had a prior influence on (i.e. was partially mediated by) DTV, with both being mediated in turn by flow of liquid. Age picked up residual variance not accounted for by these. This pattern of effects is shown in Fig 9, with the Study 1 model included for comparison. Path analysis using a maximum likelihood approach confirmed that this new model provided a high degree of fit to the data, χ^2^ = 0.953, df = 1, p = .329.

All differences between the two models reflect the methodological changes between studies. The more diverse sample of Study 2 led to a stronger relationship between vocabulary and causal performance, the age x rotation effect disappeared with the more discriminating rotation task of Study 2, and the effect of logarithmic block design was partially mediated by flow of liquid, detectible in Study 2 due to the inclusion of DTV instead of the ineffective perceptual speed task of Study 1. There was also now a strong link between DTV and flow of liquid. Replicated in both models are strong direct effects on causal performance of nonverbal ability and, most importantly, spatial-temporal analysis.

#### 3.2.3 Identification of causal mechanisms

Chi-square analyses were used to examine the specific relationship of flow of liquid to inference of mechanisms, though instances of these were less frequent. Table 10 shows the percentage of children obtaining perfect scores for flow of liquid at each level of explanation score for sinking, absorption and solution. The pattern here was clearer than for Study 1, assisted by the refinement of the scoring system. For all three processes, performance on flow of liquid was strongly associated with coordination of variables, and the step on from there to mechanism was always made by those who 'got' it.

**Table 10:** Percentages of children (N = 124) obtaining perfect scores on the flow of liquid task at each level of explanation response.

	Inference score				
Causal task	4 (mechanism)	3 (coordinated factors)	2 (uncoordinated factors)	1 (single factor)	0
Sinking	100.0	97.3	52.5	50.0	30.8
Absorption	100.0	92.9	69.2	46.1	21.4
Solution	100.0	94.1	73.3	41.2	15.8

While perfect performance on flow of liquid did not guarantee coordination of variables or inference of mechanism, the percentages of children who exhibited such performance declined sharply below the level of coordination responses; if they failed to grasp flow of liquid, therefore, they were unlikely to have achieved coordination, let alone inference of mechanism. The chi-square results for explanation score by perfect versus non-perfect flow of liquid score were significant for sinking, χ^2^ = 31.496; absorption, χ^2^ = 21.882; and solution, χ^2^ = 38.717; df = 4, p < .001 for all. All of those who gave any mechanism responses had a perfect flow of liquid score; of those who gave none, it was 59.0%, Fisher's exact test, p < .001.

A similar pattern was apparent for DTV. Children with coordination and mechanism responses consistently had significantly higher scores on this measure (see S5 Appendix in the supporting information), as did those who made any mechanism responses compared to those who did not, mean = 15.89 vs. 13.11, t(1,122) = 5.025, p < .001. This confirms that higher level causal inference and awareness of invisible mechanisms is strongly related to spatial-temporal analysis as indexed by both flow of liquid and DTV.

### 3.3 Discussion

Study 2 confirmed and extended the key findings of Study 1. First, as before, children gave accurate descriptions of what they observed in the causal tasks, but their ability to predict, justify and explain what they witnessed–especially as regards mechanisms–improved more slowly with age. However, a reasonable percentage were able to give explanation responses that explicitly coordinated important dimensions of variables.

Second, ability to analyse spatial-temporal transformations also improved over the age range, with children exhibiting similar performance on both flow of liquid and the new DTV tasks.

Third, taken on its own, the DTV spatial-temporal measure was a good predictor of explanation and overall causal performance, and consistently predicted higher levels of causal inference–both coordination of variables and reporting of mechanisms. It also shared its variance with flow of liquid–the stronger predictor–when that was included. This suggests that the common element of both tasks, spatial-temporal analysis, was the primary source of the influence of flow of liquid on causal performance.

The spatial measure, rotation, had no predictive value, despite being similarly discriminating.

In this more diverse sample, vocabulary—generic verbal ability, was also a strong predictor of performance overall. Nonverbal ability had a weaker direct influence, but had an additional effect via its impact on spatial-temporal ability.

## 4. General discussion

The two studies presented here differed in terms of sample characteristics and the implementation of the causal and other tasks, yet produced highly consistent results. These provide important insight into the development of children’s capacity to make inferences about continuous causal processes and unobservable mechanisms; and in particular clarify the role in this of verbal, nonverbal, spatial and spatial-temporal analysis. Table 11 summarizes the similarities and the differences between the two cohorts.

**Table 11:** Characteristics of the two cohorts.

	*Study 1 (N = 107)*	*Study 2 (N = 124)*	*Significant effects*	
			*Study 1*	*Study 2*
*Hypothesis*	Spatial-temporal analysis provides a bridge between observation of continuous processes and their causal analysis.			
*Sample characteristics*	Middle and high SES	Low and middle SES		
*Implementation of causal phenomena*	A three stage implementation (prediction, description, explanation)	A five stage implementation (observation, prediction, justification, testing, explanation)		
*Spatial-temporal tasks*	Flow of liquid	Flow of liquid	✔	✔
	Speed	Distance, time, velocity	X	✔
*Spatial tasks*	Monkey mental rotation	Mental rotation (Tick-Tock)	✔ for young children	X
	Paper folding	-	X	
*Verbal task*	WASI vocabulary	WASI vocabulary	X	✔
*Nonverbal task*	WASI block design	WASI block design	✔	✔

### 4.1. The development of causal reasoning about continuous processes

Both studies provided consistent evidence that primary schoolers have the ability to think about continuous natural processes, and also demonstrated that constraints on their performance are not a function of their immediate prior exposure to instances of these. More direct experience, as in Study 2, clearly does not guarantee performance improvements, even at this basic level of science reasoning. The different task components varied in difficulty, however: around 80% of children across all ages, tasks, and in both studies made and reported completely accurate observations. For prediction, the percentage decreased to just under 50% overall. Explanation scores trailed, with only about 15% reaching top scores.

Predictions in Study 1 were made ahead of observation, so required prior knowledge either consolidated from past observations or others’ reports, plus recognition of how this applies to the present context. This was found to be more demanding than description of current observation. The sizable difference in performance suggests many children lacked well-developed existing knowledge for any of the three processes. In Study 2, prediction scores were made after initial observations to inform prediction, but performance remained poor. This could reflect sample differences, and also the increased complexity of the task.

As compared to prediction and description, children’s explanations were the lowest at all ages in both studies, and mechanism responses were relatively unusual. In Study 1, only 20.6% of children, the majority in the oldest age group, identified an underlying causal mechanism, with 5.6% doing so across more than one phenomenon; the lower corresponding values in Study 2 were 15.3% and 1.6%, again consistent with the more socially mixed sample. Across both studies, children’s explanations were typically limited to abstracting causal variables from the observed objects. At the same time, the more extended range of examples employed in Study 2 allowed us to see that a reasonable proportion of older children had progressed not only to linking causal factors to observed effects, but also to recognising the coordinated operation of key variables, i.e., to recognizing that there is an underlying relationship. This in turn may lead into thinking of how the variables are connected to each other and to the effect, as an important precursor to thinking about mechanisms.

Mechanism inferences remained less common in contrast to past research showing that pre-schoolers are able to make inferences about mechanisms in simpler machine/toy systems [31], [45], [47], [48]. We argue that this striking lag is because continuous processes are hard for children, since they require spatial-temporal information to be analysed in different causal systems. We elaborate this in section 4.3.

### 4.2. The development of spatial-temporal analysis

The novel tasks employed to assess children’s spatial-temporal analysis all revealed reasonable performance and a regular developmental progression. Study 1 considered a highly analytical and a highly perceptual spatial-temporal task, speed, with very different processing characteristics.

Flow of liquid involved a coordinated spatial-temporal transformation in a single trial, slowed down and segmented by the experimenter, who stopped the flow at successive states to prompt children for accurate observation and depiction of each. If this form of analysis of sequential states is helpful, it might enable them to extract the principle that as the top level decreases over time, the bottom liquid level increases, and to reconstruct the sequence after the link between top and bottom was cut. Consistent with this, children with perfect scores tended to solve the task quickly, forming a decreasing/increasing sequence for the top/bottom half, then matching the bottom/top; or placing the initial and final pairs, then filling in intermediate states. Children who made errors tended to do so throughout the task, and were much slower and unsystematic at the final stage. In sum, flow of liquid involved a highly analytic response, made easy for most children by the care taken to ensure they understood how the drawings related to their observations.

The speed task, in contrast, had no equivalent support. It involved 17 trials, each with a brief spatial transformation unfolding over 4–6 seconds; children responded rapidly, within a few seconds. Thus, the two tasks required very different processing. The average difficulty level of this particular perceptual task was clearly higher than that of the analytical flow of liquid task, however, which might be attributable to various contrasting features. The three bunny objects in the speed task moved entirely independently, while the states of the top and bottom flask were linked. Thus, accurate responses in the speed task depend on processing the successive spatial states for each object, while both logical and causal constraints may support correct responding in the flow of liquid task. It is also possible that sequencing the past is inherently easier than predicting the future (even in deterministic systems), simply because the latter is less supported by past experience. Finally, the speed task was a computer task involving a 2-dimensional display, while flow of liquid involved a live demonstration involving 3-dimensional objects, and this may have supported performance, as in other task domains [68], [69].

We also consider it plausible, though, that the different processing characteristics of the tasks were responsible for their differences in performance and predictive value. It would seem that flow of liquid involves an analytic-inferential response, while the speed task afforded rapid perceptual judgments. The former may have been relatively easy for children here, because of the care taken to get the initial observation correct, and to make clear to children how the drawn representations related to the observations. Perceptual judgement in the speed task may be difficult due to the rapid motion involved, which, to the extent that children may have tried for a more analytical approach, did not facilitate segmentation either.

As a primary purpose of this research was to ascertain if causal reasoning was predicted by spatial-temporal skills, Study 2 therefore focused on analytical spatial-temporal ability, rather than perception. Thus, the perceptual speed task was replaced with a set of tasks (DTV), but with different processing characteristics, requiring children to make mental simulations, with an accompanying explicit time count that tacitly segmented the imagined motion states. They made judgments of the distance travelled, time taken, or relative velocity of various animals under these conditions, which afforded a very different approach to the perceptual speed task of Study 1. In the distance and velocity tasks, children frequently traced the motion of the animals one by one across the screen with their finger, apparently using this as an external accompaniment to support their judgements. In the velocity task, they also commonly discounted the slowest animals (turtle, snail) as candidates at the outset. Both points are consistent with them employing an analytical spatial-temporal approach.

There were task-specific variations in the requirements of these spatial-temporal tasks as well. All the DTV tasks required children to imagine and project object movement, not needed in flow of liquid, where children simply re-ordered their drawings of a directly witnessed and explicitly segmented sequence. Moreover, although scores on the three DTV tasks were broadly similar, velocity was slightly more difficult, sharing less variance with the others, underscoring that spatial-temporal analysis may have multiple forms.

Nevertheless, performance on all DTV measures and flow of liquid showed similar increases across the age range, with most children performing well on all these tasks by Y5, indicating they were comparable in terms of difficulty. Scores on flow of liquid and DTV were also correlated in contrast to flow of liquid and perceptual speed in Study 1, even when age and general ability were controlled. This suggests a consistency with regard to DTV and flow of liquid tapping into a common underlying dimension of analytic–as opposed to perceptual–spatial-temporal ability.

One can suggest that while flow of liquid clearly involves a sequence of spatial-temporal state changes, it may draw on children’s logical ability to form a transitive, ordered series, visual-spatial working memory capacities, or knowledge of a causal process (e.g. flow under gravity, even if the response did not require any articulation of this causality). To clarify these points, further research is needed. However, for now we can highlight that none of the DTV measures relied on transitive inference or causal analyses, and the high correlations between the flow of liquid and DTV tasks can therefore be interpreted as indicating that the spatial-temporal elements of both tasks were substantially central to their overlap. We acknowledge that working memory capacity may influence performance on both tasks, given their reliance on manipulation of visual-spatial information. However, preliminary research on this by Lim [70] suggests that any such influence is a modest and peripheral one: the Corsi Block Span was found to explain a little over 10% of variance in performance on the flow of liquid task in a sample of 10 to 13 year olds, and was not predictive of causal inference.

### 4.3. What predicts children’s causal reasoning about continuous processes?

In Study 1, the spatial-temporal flow of liquid task distinguished between lower and higher levels of overall causal performance across the age range, and was the *only* predictor of higher levels of causal explanation. It was the only measure consistently sharing little variance with the other predictors, including the spatial measures, confirming its distinctness from these. This result was replicated in Study 2. Even with the extended causal task and measurement indices, the flow of liquid spatial-temporal measure remained the strongest predictor of children’s causal performance–stronger in fact overall than in Study 1, and if anything discriminating to a greater extent at the higher levels of overall causal performance.

Unlike the speed task in Study 1, the other spatial-temporal measure, DTV, designed to share analytic characteristics with flow of liquid, was also a significant predictor of overall causal performance, coordination of variables and inference of mechanism. It too explained unique variance beyond verbal, nonverbal and spatial measures, and it correlated well with flow of liquid, controlling for other factors. This clear overlap in explained variance again indicates that the DTV tasks and flow of liquid measure similar spatial-temporal competences. However, DTV did not survive the inclusion of flow of liquid in the regression models and the path analysis indicated that the influence of DTV was subsumed by its commonality with flow of liquid, while flow of liquid appears to assess a further and additionally predictive dimension. One obvious candidate for this would seem to be the ability to use spatial-temporal information to extract the underlying principle governing the pattern of change.

Of three spatial tasks across the two studies, only one, monkey rotation, predicted causal performance independently, but its influence was restricted to the youngest age group in Study 1. The other spatial measures (paper folding, Tick and Tock) were not related to overall causal performance or to explanation/mechanism. We acknowledge that spatial ability may take various forms, but its role in causal reasoning about continuous processes seems limited, and appears to overlap in particular with verbal and also nonverbal ability (contrary to e.g. Paivio [71]), with which it shared variance in both studies. This lack of predictive power might be due to spatial tasks relying on two-dimensional representations and not carrying temporal information. Missing the temporal element may cause them to be encoded in a discrete fashion instead of on-line spatial representations. Discrete transformations seem not to provide feedback for the past-current-future representations either (see Kosslyn [72, 73], for the argument on the characteristics of spatial representations being quasi-pictorial). That the spatial measures consistently shared their variances with verbal ability may suggest that mapping spatial properties into language is inherent in semantic representations (see [74], on the role of natural language in spatial memory when using geometric and non-geometric information to relocate the body; see also [36, 75]).

Nonverbal ability, in contrast, as indexed by block design, did predict causal reasoning independently of spatial-temporal ability. However, its influence was consistently restricted to discriminating at lower levels of causal performance. This may be because it involves the detection and analysis of spatial–but not spatial-temporal–patterns (cf. the identification of factors and variables), which is then superseded by spatial-temporal analysis as driver of later causal reasoning developments.

Verbal ability, as indexed by vocabulary, did not predict causal performance in Study 1, which had a quite homogenous sample with relatively high socioeconomic background (nonverbal ability was more discriminating in this case), but it became prominent in Study 2, when the gap between high and low income families and school catchment areas widened. However, vocabulary was not associated with the parental indices in Study 2, and the effect was not attributable to the sample exhibiting a greater spread of verbal ability: comparison of the means and standard deviation across the two samples shows that Study 1 verbal scores clustered in the middle of the scale, and were similar to those of children in Study 2, possibly due to schools equalizing verbal ability.

The key difference between the two samples was that in Study 2, children’s nonverbal ability was notably worse–unexpectedly so for children of better educated parents, but perhaps again reflecting schooling. This led to verbal ability having greater importance for causal performance. This suggests that it is nonverbal ability that plays the major role as a foundation for spatial-temporal ability, in line with the Study 2 path model. It also suggests that there is some compensatory relationship between nonverbal and verbal ability, explaining why vocabulary was as strong a predictor of the causal indices as flow of liquid. Rather than a wider effect of social area and home environment then, it may be the relationship between verbal and nonverbal ability that matters. This plainly merits further investigation.

**Fig 10:**
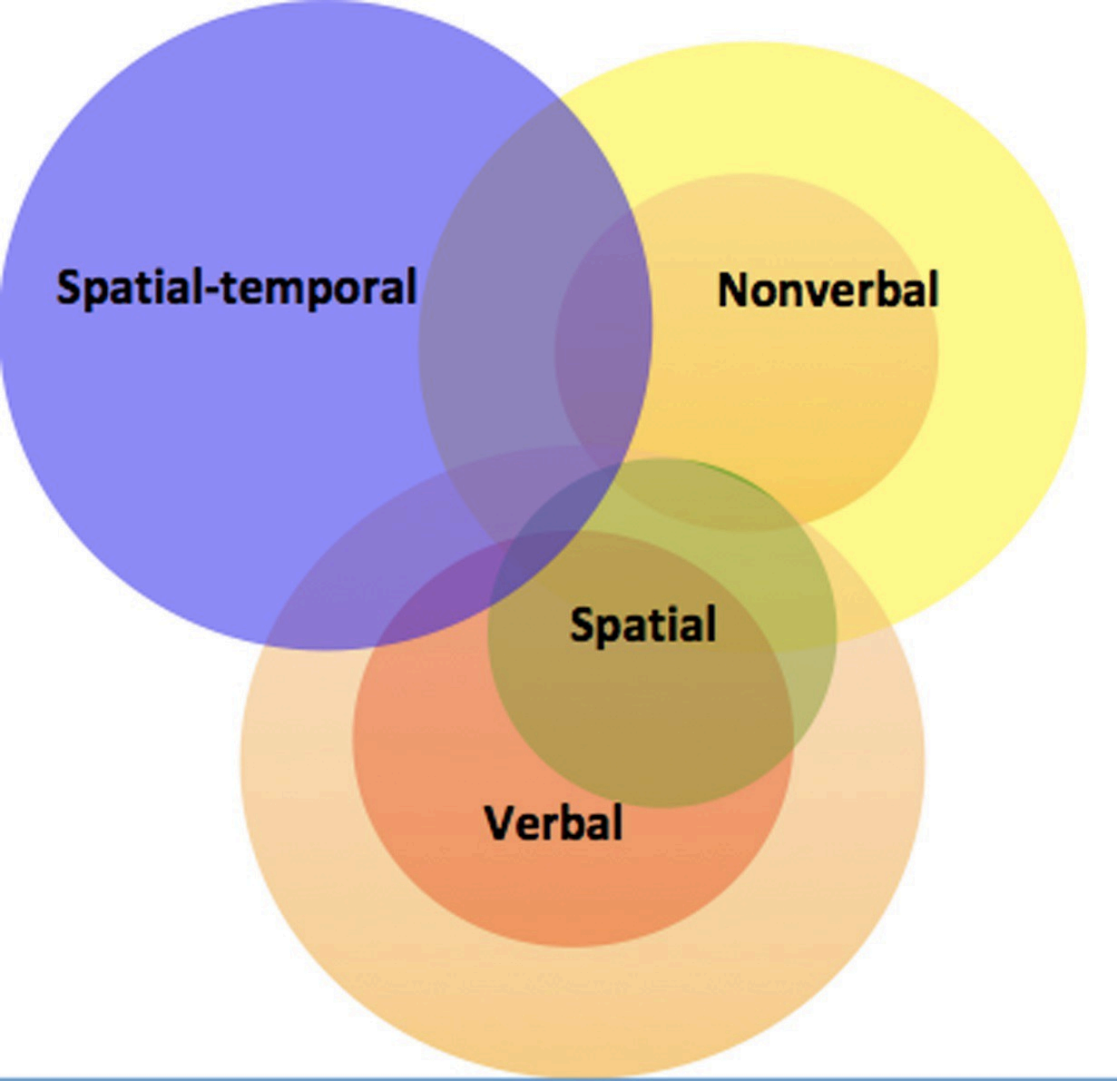
Schematic showing the relationship between spatial-temporal, spatial, verbal and nonverbal ability in the prediction of causal reasoning.

The conceptual map in Fig 10 illustrates these relationships in task variance, including the variable and compensatory influence of verbal and nonverbal ability. Spatial ability is subsumed within verbal and nonverbal ability, and has no similar compensatory relationship with spatial-temporal ability.

### 4.4. The cognitive processes involved in children’s spatial-temporal analysis

Consideration of the characteristics of the spatial-temporal tasks and the findings related to them provides further insight into the nature and development of spatial-temporal analysis. For instance, flow of liquid focused on the segmentation and organisation of time-dependent spatial changes, and the capacity to capture these enabled children to move beyond simple perception of continuous causal processes. The DTV tasks provided or asked for an explicit time count that similarly allowed children to mentally simulate and project the movement of a set of objects, coordinating and integrating information about other given dimensions. This requirement has clear parallels to the demands of thinking about differences in speed in instances of continuous causal processes. In both cases though, segmentation per se is not the only issue: upstream spatial-temporal skills also matter. These include coordination of spatial-temporal information, projection of movement and extraction of underlying principles governing time-related changes, but this is unlikely to be an exhaustive list.

There seem to be multiple forms of spatial-temporal thinking, therefore, but the ability to extract these kinds of information about processes from their spatial and temporal qualities is established within the primary years. However, relatively few children were able to identify mechanisms in the causal tasks, while the ability to coordinate variables appeared to be an important interim step. Thus, the ability to utilize spatial-temporal information in causal processes may develop more slowly.

A key issue in this may be that time is continuous, and extracting temporal information continuously is difficult. By slowing down and segmenting temporal processes it becomes possible to analyse and reflect on temporally ordered change, including construction of images and ideas about transformation–a kind of analysis not captured by spatial tasks, and one which would facilitate analysis of invisible properties (e.g. force or density), as crucial in causal reasoning.

In applying spatial-temporal ability to causal processes, though, children still need to construct the actual content of ideas about mechanisms. Successful analysis may require one to utilize the following steps, across successive experiences:

1. Extraction of information from object states over time (perceptual)
2. Utilizing multiple forms of representation to capture important aspects of change in state-time (representational)
3. Analysis of representations, according to task requirements (analytical):

3a extraction of the regularity captured by the sequence

3b abstraction of causal relations linking object features to effects, including integration and coordination of operative variables

1. Further inference, possibly by mental imaging (cf. [28, 76, 77]), of a mechanism to link these causal object features to the effects (imagery).
2. Evaluation against observed change over time to modify/confirm the enriched segmented representations (feedback)

The data in both studies provide clear evidence that typical development in the primary age range covers the first three steps. Only some children manage to get to the fourth. Although we did not directly test the fifth, there were signs that those children who showed advanced causal understanding employed active feedback for their representations, adjusting their ideas between the prediction and explanation stages in the Study 2 causal tasks.

## 5. Conclusion

This is the first research examining the role of spatial-temporal analysis in children’s reasoning about continuous causal processes, and their inferences of the invisible mechanisms that underlie these. Children showed increasing capacity for spatial-temporal cognition–distinct from spatial cognition–during the primary school period. This spatial-temporal analysis is a key driver of their causal cognition in continuous processes, though it would appear to have multiple forms that may support different aspects of mechanism inference, and the application of these to causal analysis only gradually emerges. Given the importance of the analysis of continuous causal processes in science and everyday life, more research is needed to examine further the nature and role of spatial-temporal ability in this.

## Supporting information

S1 Appendix Empirical detail from [50]: Children’s reasoning about continuous causal processes: The role of verbal and nonverbal ability. (DOCX)

S2 Appendix Hierarchical regression analyses for causal components. (DOCX)

S3 Appendix Note on prediction materials and scoring in Study 2. (DOCX)

S4 Appendix Study 2 scoring system and examples. (DOCX)

S5 Appendix Mean DTV scores at each level of explanation response. (DOCX)

S1 Data Minimal data set Study 1 data. (XLSX)

S2 Data Minimal data set Study 2 data. (XLSX)
