# Supplementary material for: The role of spatial and spatial-temporal analysis in children’s causal cognition of continuous processes
Source: PLoS One. 2020 Jul 30;15(7):e0235884. doi: 10.1371/journal.pone.0235884 (PMC7392260; doi:10.1371/journal.pone.0235884)
Supplement: S1 Appendix — (DOCX) [file pone.0235884.s001.docx]

**Method**

The three *causal tasks* each highlighted a direct contrast between two instances of the target phenomenon, presented simultaneously. For *sinking*, children saw a stone and a blueberry of similar size but different densities, which sank at different rates in a large jar of water. For *absorption*, they saw water rising from a petrie dish through strips of tissue and blotting paper of the same length/width, the water rising faster through the more open structure of the tissue. For *solution*, children saw the same small quantities of table and rock salt dissolve in warm water, the greater surface area to volume of the table salt leading to more rapid solution (see Figure S1.1).

| Tasks | Materials | |
| --- | --- | --- |
| Sinking  (stone, blueberry)  Absorption  (blotting paper, tissue paper)  Solution  (table salt,  rock salt) | *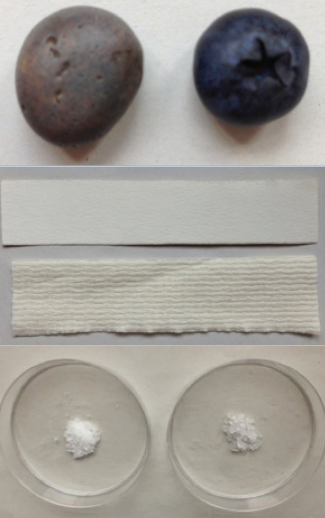* | |
|  | |  |
|  | |  |

Figure S1.1. Materials used in the causal tasks.

Each task had the same three-stage structure, in which children: (1) inspected the contrasting materials and were asked what they thought would happen when they were put into the water (prediction from *prior knowledge*); (2) watched the focal events and were asked to *describe* what they had noticed; (3) were asked to *explain* why they thought things had happened in the way that they had seen. At each stage, they were encouraged to give as full an answer as they could (e.g. ‘do you think the same thing will happen to both?’; ‘did you notice anything else?’; ‘do you think there might be another reason?’).

Responses were given initial scores for prior knowledge, description and explanation as children answered (see Table S1.1 for scoring system). Prior knowledge and description were scored for accuracy of anticipating/reporting differences in sinking/absorption/solution rate. Explanation scoring began at the minimal level of the observed factor(s) (score of 1); via making explicit that these are variables linked to the observed differences in speed of the contrasting examples (score of 2); to a statement about the underlying mechanism which produced the effect (score of 3). Table S1.2 shows examples of responses at each level of explanation for each task.

Table S1.1. Scoring system for causal tasks.

| **Component** | **Sinking** | **Absorption** | **Solution** |
| --- | --- | --- | --- |
| Prediction from *prior knowledge*  (0-2) | Correct prediction for stone (i.e. sinks) =1  Correct prediction for difference between stone and berry (i.e. sink at different speeds)=1 | Correct prediction for tissue paper=1  Correct prediction for difference between tissue and blotting paper =1 | Correct prediction for table salt=1  Correct prediction for difference between table and rock salt=1 |
| *Description* of observation  (0-2) | Correct description for stone=1  Correct description for berry=1 | Correct description for tissue paper=1  Correct description for blotting paper=1 | Correct description for table salt=1  Correct description for rock salt=1 |
| *Explanation*/  inference  (0-3) | No/irrelevant explanation=0  Weight/size without difference between objects=1  Weight/size with difference=2  Density and mechanism=3 | No/irrelevant explanation=0  Thickness/softness/texture etc. without difference between types of paper=1  Thickness/softness/texture etc. with difference=2  Nature of papers/holes and mechanism=3 | No/irrelevant explanation=0  Grain/size etc. without difference between types of salt=1  Grain/size etc. with difference=2  Grain/size etc. with surface area and mechanism=3 |

To confirm reliability, two researchers subsequently scored all responses independently from the audio-recordings. Agreement was 93%, and final scores were assigned following discussion and checking the audios in the small number of instances where there was a difference.

Table S1.2. Examples of explanation responses

| Phenomena | Level 1 | Level 2 | Level 3 |
| --- | --- | --- | --- |
| Sinking | “They are heavy and they sank to the bottom” | ‘The stone is heavier than the berry so they sank to the bottom differently” | “They are both heavier than the water and cannot hold air in it so they sank to the bottom. But the stone sank quicker than the berry because it’s got more stuff in it so the water can’t hold it up as it did to berry.” |
| Absorption | “If you dip the paper in the water they get wet because they’re soft” | “The tissue paper is thinner than the other paper so water rises faster in it” | “The tissue paper has holes in it that help water to rise up. Water holds on the walls of the holes and layers and that helps it to climb up. Other paper has some space in it, but not as much as the tissue paper.” |
| Solution | “They go into water because they’re small and spread out” | “The table salt is smaller than the rock salt so it disappears quicker.” | “The size of the two types of salt is different. And this is more rocky so water cannot go into it easily. They both dissolve in the water, but rocky one takes more time than the table salt.” |

Composite scores were computed for each task (0-7), for each response component (0-6 for prior knowledge, description, 0-9 for explanation), and for number of mechanism level responses across tasks (0-3).

**Results**

*Causal task performance*

*Tasks.* Figure S1.2 shows the response profiles for each age group on the sinking, absorption and solution tasks. Performance was best on sinking, followed by absorption, with solution some way behind. A two-way mixed ANOVA (task within-subjects, age between-subjects) found significant main effects of task, F(2,208)=47.202, p<.001, partial eta-squared=.312, and age group, F(2,104)=24.250, p<.001, partial eta-squared=.318; and a modest task x age interaction, F(4, 208)=5.056, p=.001, partial eta-squared=.089, reflecting the greater growth on solution between Y1 and Y3.


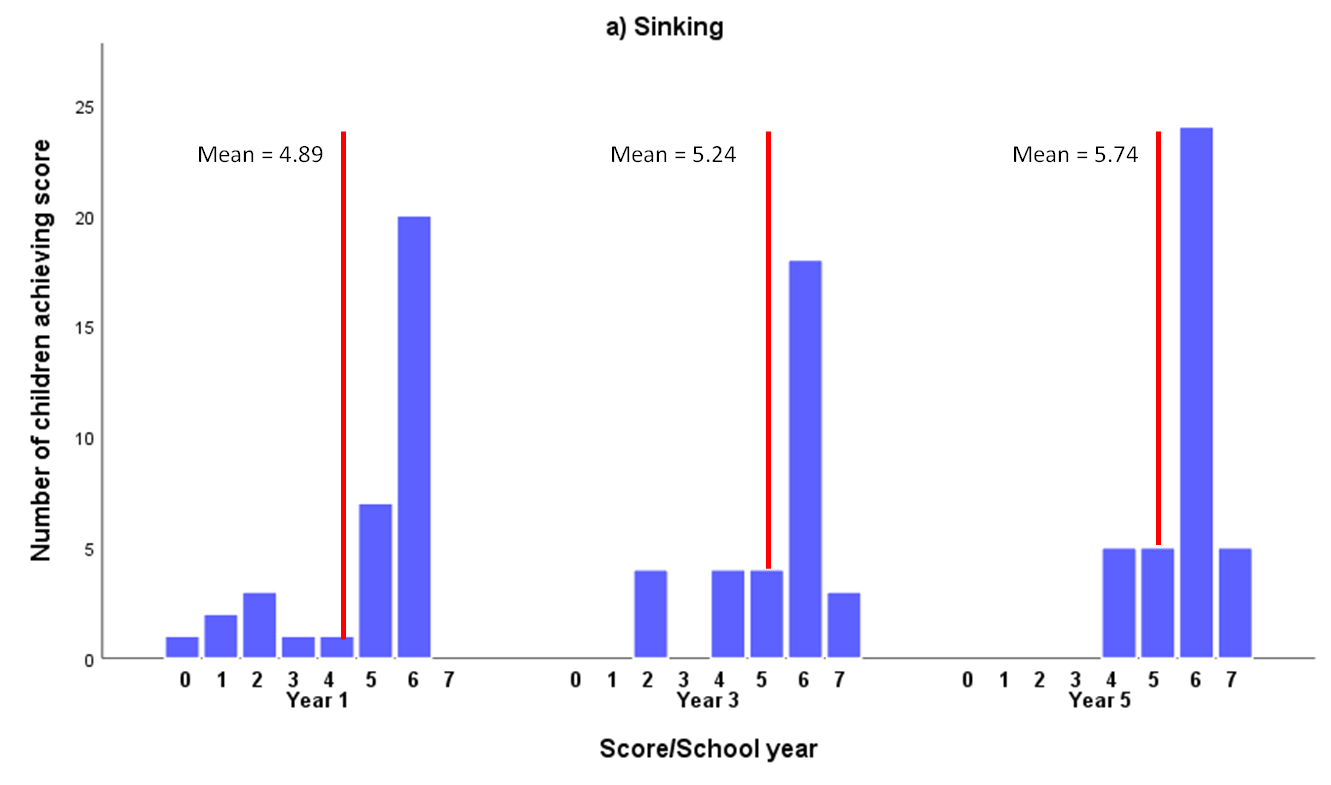


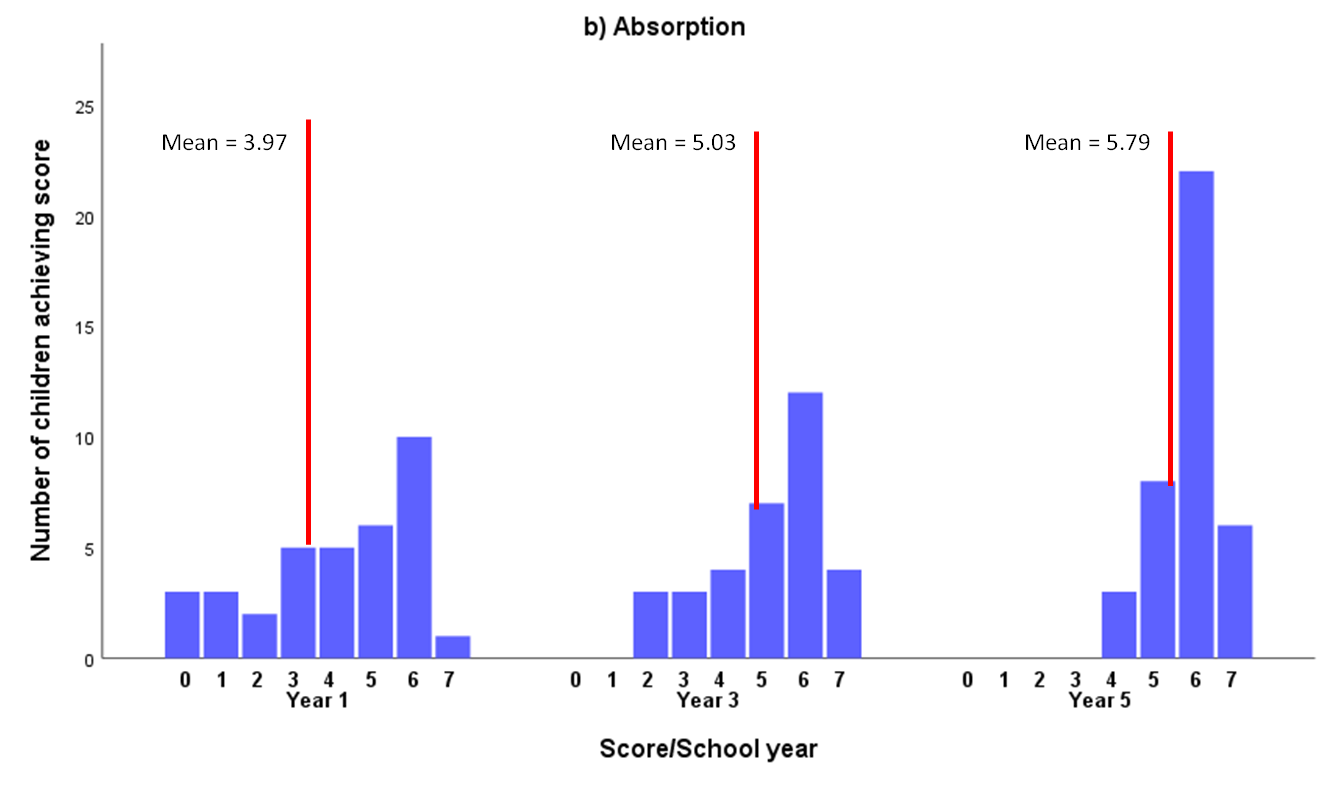


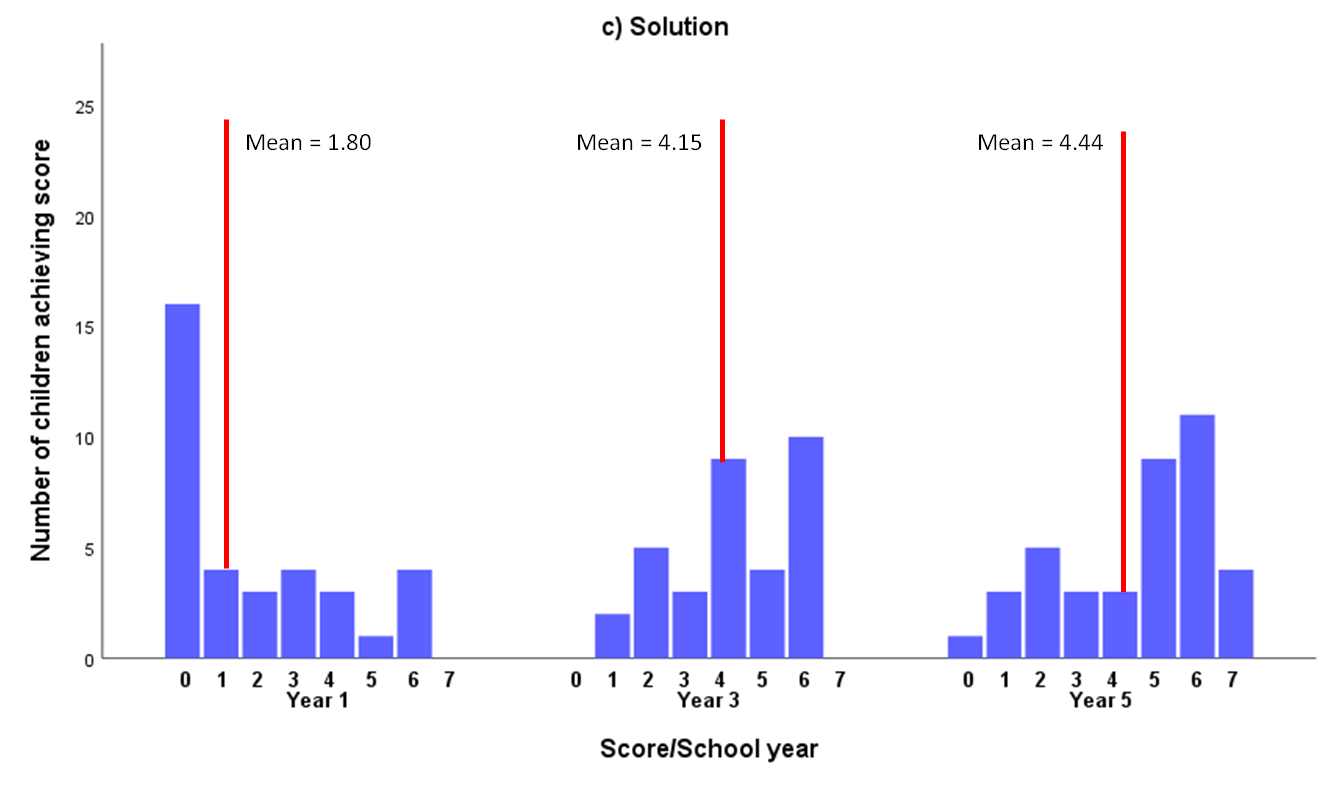


Figure S1.2. Profile of scores on a) sinking; b) absorption; and c) solution (max = 7).

*Causal components.* The profiles of each age group for prior knowledge, description and causal explanation are shown in Figure S1.3. Children performed at a high level on description, slightly less well on prior knowledge, and at a notably lower level on causal explanation. For *description*, 91.6% of children obtained the maximum score in the sinking task, 85% in absorption, and 57.9% in solution. For *prior knowledge*, the corresponding values were 72.9% for sinking, 62.6% for absorption, and 37.4% for solution.


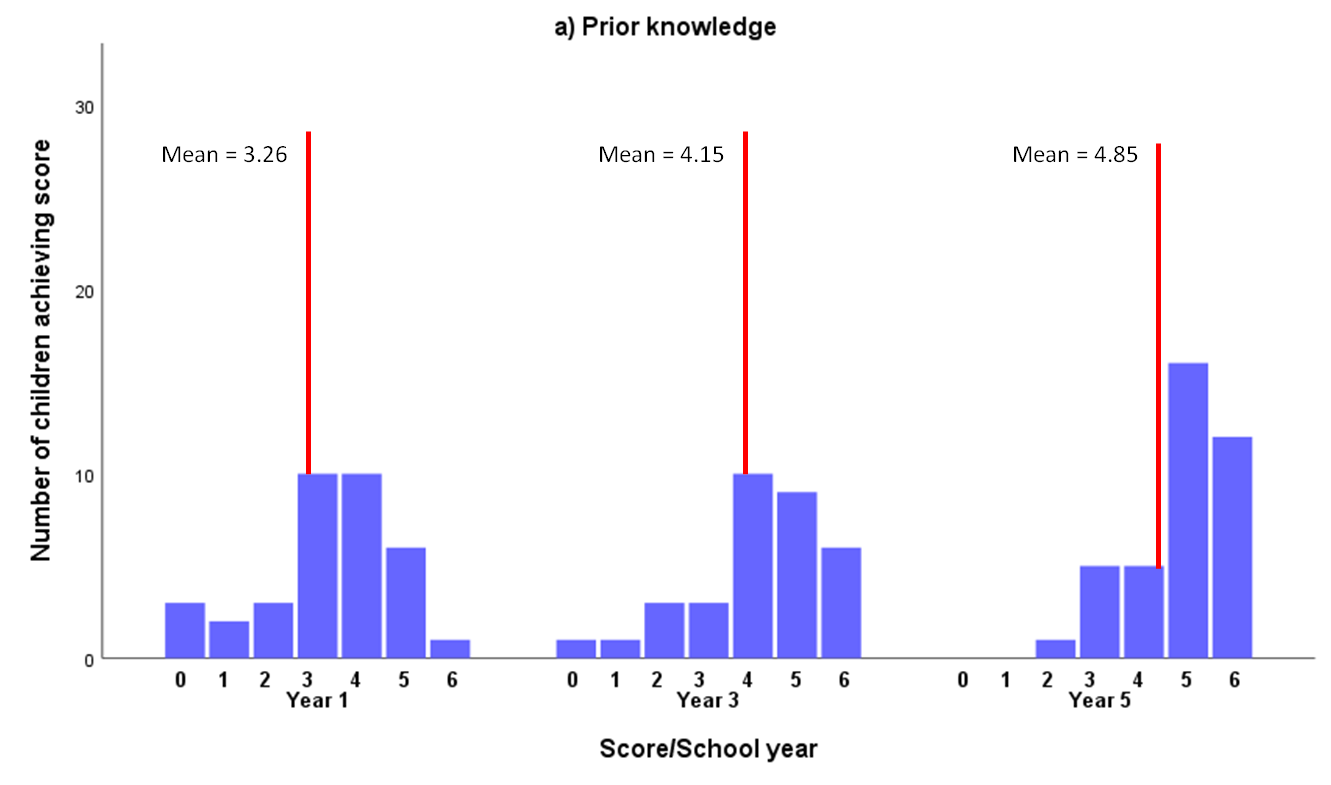

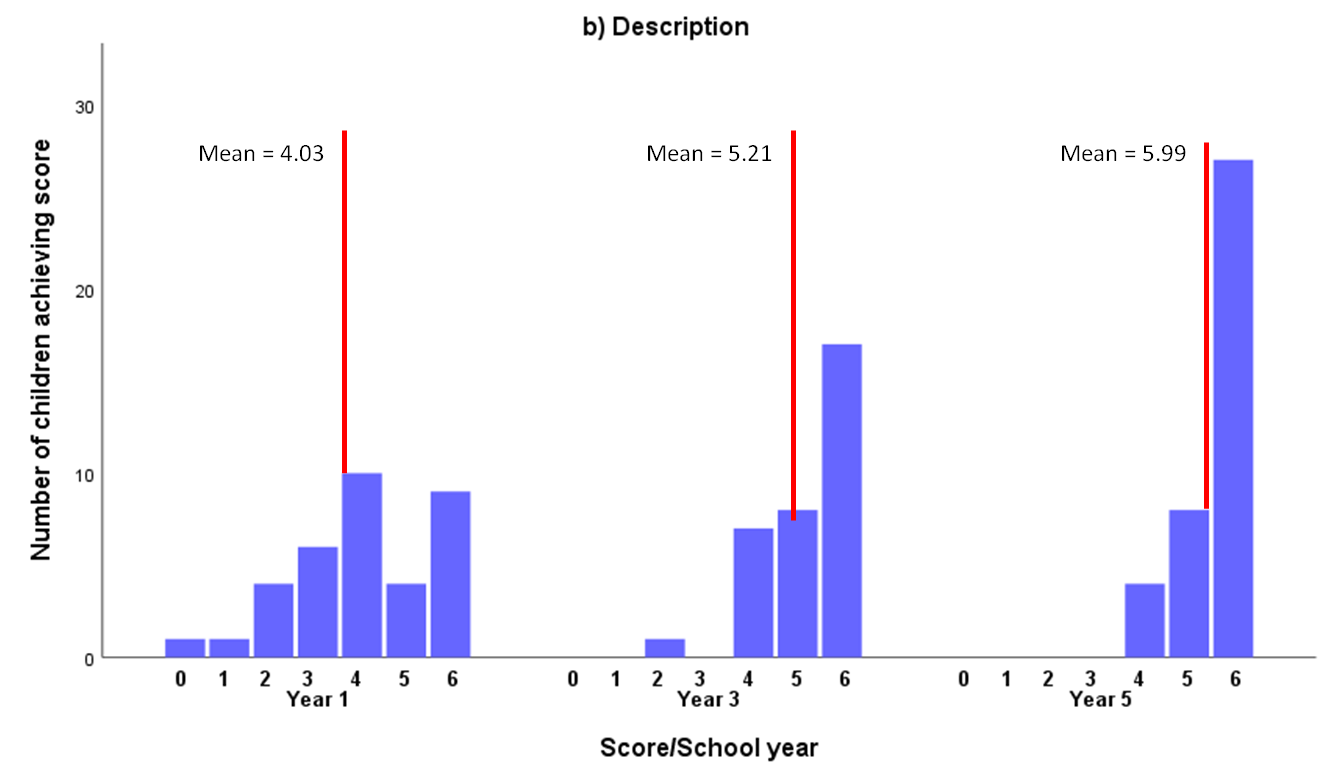


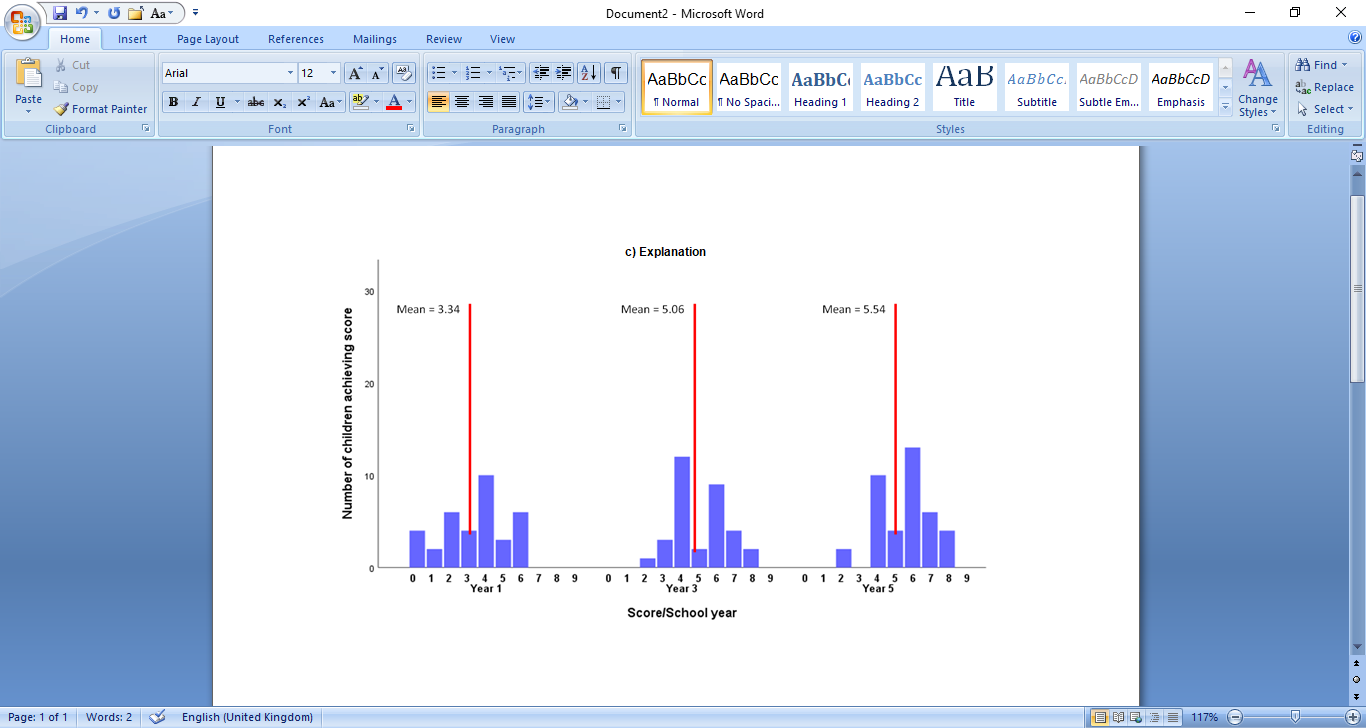


Figure S1.3. Profile of scores on a) prior knowledge (max = 6); b) description (max = 6); and c) causal explanation (max = 9).

In contrast, for *explanation*, only 9.3% got the highest score in sinking, 14% for absorption, and 4.7% for solution. The majority of explanation responses on all three tasks focused solely on identification of causal factors or variables (scores of 1 or 2). Although mechanism responses became more common in the two older groups (for sinking, there were 0 in Y1, 4 in Y3 and 5 in Y5; for absorption, 1, 6 and 8 respectively, and for solution, 0, 0 and 5), children apparently found it difficult to make the shift to this level of thinking. However, if they made any reference to mechanism at all, they tended to do so on more than one task, at over 2.5 times the chance rate, so the shift appeared to be domain-general when it occurred.

Direct statistical comparisons between the components were not made, given differences in the scales and dimensions measured. However, one-way ANOVAs showed age-related progression on each: for *prior knowledge*, F(2,104)=12.376, p<.001, partial eta squared=.192; for *description*, F(2,104)=18.336, p<.001, partial eta squared=.261; for *explanation*, F(2,104)=17.383, p<.001, partial eta squared=.251. In each case, there were significant differences between Y1 and Y3, but not between Y3 and Y5. The components were positively correlated with each other, controlling for age (for *prior knowledge* and *description*, r=.392; for *prior knowledge* and *explanation*, r=.414; for *description* and *explanation*, r=.618, all p<.001).

*Is children’s causal reasoning associated with verbal and nonverbal ability?*

There was significant positive skew on block design, due to the oldest age group having a longer tail. Vocabulary was normally distributed. One-way ANOVAs found significant increases with age on both, however: for vocabulary, Welch robust statistic=54.093 (df = 2, 67.790); for block design, 45.070 (2, 63.948), p<.001 for both, with significant differences between all three age groups. Variance was not notably attenuated for either measure: for vocabulary, overall mean=29.95, sd=7.586; for block design, overall mean=22.23, sd=13.860.

Table S1.3. Zero-order and partial correlations between causal reasoning, verbal and nonverbal ability (significant associations in bold).

|  |  | Prior | Description | Explanation | WASI vocab | Block | Block (log) |
| --- | --- | --- | --- | --- | --- | --- | --- |
| Prior |  | **1** | **.518^***^** | **.531 ^***^** | **.466 ^***^** | **.473 ^***^** | **.555 ^***^** |
| Description |  | **.392^***^** | **1** | **.703^***^** | **.439^***^** | **.391^***^** | **.476 ^***^** |
| Explanation |  | **.414^***^** | **.618^***^** | **1** | **.467^***^** | **.441^***^** | **.516 ^***^** |
| WASI vocabulary |  | **.265^**^** | .150 | **.224^*^** | **1** | **.677^***^** | **.679^***^** |
| Block design |  | **.286^**^** | .104 | **.195^*^** | **.416^***^** | **1** | **.923^***^** |
| Block design (logarithmic) |  | **.408^***^** | **.241^*^** | **.315^***^** | **.431^***^** | **.867^***^** | **1** |

Zero-order correlations above diagonal, N=107; partial correlations below diagonal, N=106 due to missing date of birth data for one participant; *p<.05, **p<.01, ***p<.001

*Correlations between variables*. Zero-order Pearson correlations showed the three causal components were positively associated with both vocabulary and block design scores, which were themselves positively correlated with each other (Table S1.3). The relationship of block design to the causal measures was logarithmic, and log block design (the logarithmic transform) was more strongly correlated with these than the untransformed score. When age in months was controlled for, log block design showed a stronger correlation with the components than vocabulary, which was uncorrelated with description responses. Parental occupation and education correlated with each other, r=.609, but otherwise only with nonverbal ability, .261, p=.008 and .382, p<.001 respectively, and are not considered further.

*Hierarchical regression models*. Hierarchical regressions examined the unique variance accounted for by verbal and nonverbal ability, given the association between them. Taking causal component scores and number of mechanism responses as the dependents, age in months was entered in the first, WASI vocabulary in the second and log block design at the third stage, to assess whether their effects were distinct from each other.

This analysis produced significant models and final adjusted R-square in all four analyses (Table S1.4). Vocabulary was a significant predictor at the second stage for prior knowledge and explanation, but not for description, confirming the partial correlation; or for mechanism responses. The inclusion of log block design consistently led to both age and vocabulary dropping out, leaving it the only predictor, with one exception – description, where age remained significant.

Table S1.4. Hierarchical regression analysis with component causal scores and mechanism responses as dependent variable (significant predictors in bold).

|  | *Model* | *M1* | *M2* | *M3* |
| --- | --- | --- | --- | --- |
|  | Predictor | β | | |
| Prior knowledge | Age in months | **.424^***^** | .197 | .059 |
|  | WASI vocabulary |  | **.330^**^** | .136 |
|  | Block design (log) |  |  | **.425^***^** |
| AdjRsquare = .304; *ΔR^2^* = .180^***^ for M1; .057^**^ for M2; .087^***^ for M3 | | | | |
| Description | Age in months | **.502^***^** | **.379^**^** | **.300^*^** |
|  | WASI vocabulary |  | .178 | .067 |
|  | Block design (log) |  |  | **.243^*^** |
| AdjRsquare = .277; *ΔR^2^* = .252^***^ for M1; .017 for M2; .028^*^ for M3 | | | | |
| Explanation | Age in months | **.472^***^** | **.285^*^** | .185 |
|  | WASI vocabulary |  | **.272^*^** | .132 |
|  | Block design (log) |  |  | **.307^*^** |
| AdjRsquare = .287; *ΔR^2^* = .223^***^ for M1; .039^*^ for M2; .045^*^ for M3 | | | | |
| Mechanism | Age in months | **.284^**^** | .191 | .088 |
|  | WASI vocabulary |  | .135 | -.010 |
|  | Block design (log) |  |  | **.317^*^** |

AdjRsquare = .114; *ΔR^2^* = .081^**^ for M1; .010 for M2; .048^*^ for M3.

^*^*p* <.05. ^**^*p*<.01. ^***^*p*<.001.

Path analysis using a maximum likelihood approach was employed to test the fit of the regression model for mechanism responses, treating age as a background influence. The model in which the influence of vocabulary was entirely mediated by log block design (Figure S1.4) provided the best fit to the data, chi-square=0.007, df=1, p=.932. A further mediation-moderation analysis confirmed these effects, showing that there was no interaction between vocabulary and blocks (p>.05), and that there was full mediation: the direct effect of vocabulary was non-significant, b=.00, SE=.01, t=.24, p=.81 with a small unstandardized indirect effect b = .02, CI=.01 to .05, standardised effect=.24, indicating the path model was robust.


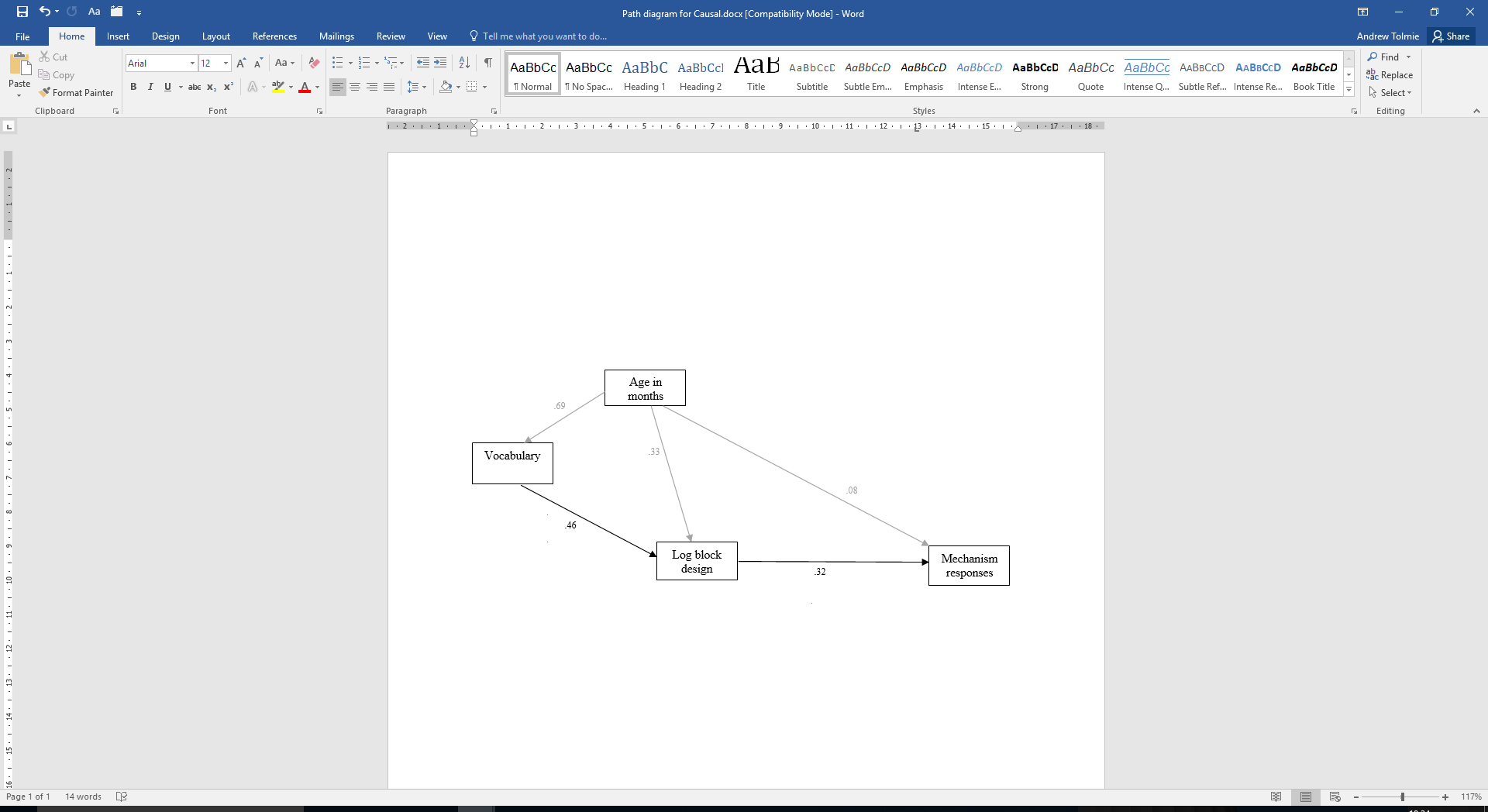


Figure S1.4. Path model including standardised coefficients for the effects of age, vocabulary and log block design on mechanism responses (subsidiary relationships in grey).
