# Supplementary material for: The role of spatial and spatial-temporal analysis in children’s causal cognition of continuous processes
Source: PLoS One. 2020 Jul 30;15(7):e0235884. doi: 10.1371/journal.pone.0235884 (PMC7392260; doi:10.1371/journal.pone.0235884)
Supplement: S2 Appendix — (DOCX) [file pone.0235884.s002.docx]

***2.1 Study 1***

*2.1.1 Prior knowledge*

| Model | M1 | M2 | M3 | M4 | M5 |
| --- | --- | --- | --- | --- | --- |
| Predictor | β | | |  |  |
| Age in months | .197 | .059 | .015 | -.002 | -.034 |
| WASI vocabulary | **.330^**^** | .136 | .120 | .099 | .061 |
| Block design (log) |  | **.425^***^** | **.351^*^** | **.324^*^** | **.292^*^** |
| Rotation |  |  | -.012 | -.016 | -.004 |
| Paper-folding |  |  | .163 | .167 | .126 |
| Speed |  |  |  | .119 | .113 |
| Flow of liquid |  |  |  |  | **.240^*^** |

AdjR^2^ = .345; *ΔR^2^* = .238^***^ for M1; .087^***^ for M2; .010 for M3; .011 for M4; .043^*^ for M5. ^*^*p* <.05. ^**^*p*<.01. ^***^*p*<.001.

*2.1.2 Description*

| Model | M1 | M2 | M3 | M4 | M5 |
| --- | --- | --- | --- | --- | --- |
| Predictor | β | | |  |  |
| Age in months | **.379^**^** | **.300^*^** | **.229^*^** | **.228^*^** | .197 |
| WASI vocabulary | .178 | .067 | .016 | .015 | -.015 |
| Block design (log) |  | **.243^*^** | .044 | .042 | .014 |
| Rotation |  |  | **.184^*^** | **.183^*^** | **.191^*^** |
| Paper-folding (log)^+^ |  |  | **.372^***^** | **.372^***^** | **.336^***^** |
| Speed |  |  |  | .008 | .003 |
| Flow of liquid |  |  |  |  | **.194^*^** |

AdjR^2^ = .421; *ΔR^2^* = .269^***^ for M1; .028^*^ for M2; .135^***^ for M3; .000 for M4; .028^*^ for M5. ^*^*p* <.05. ^**^*p*<.01. ^***^*p*<.001. ^+^ The relationship of paper-folding to description was logarithmic.

*2.1.3 Explanation*

| Model | M1 | M2 | M3 | M4 | M5 |
| --- | --- | --- | --- | --- | --- |
| Predictor | β | | |  |  |
| Age in months | **.285^*^** | .185 | .158 | .153 | .126 |
| WASI vocabulary | **.272^*^** | .132 | .117 | .111 | .080 |
| Block design (log) |  | **.307^*^** | .244 | .237 | .211 |
| Rotation |  |  | .146 | .145 | .155 |
| Paper-folding |  |  | .033 | .034 | .001 |
| Speed |  |  |  | .032 | .027 |
| Flow of liquid |  |  |  |  | **.196^*^** |

AdjR^2^ = .309; *ΔR^2^* = .262^***^ for M1; .045^*^ for M2; .018^***^ for M3; .001 for M4; .029^*^ for M5. ^*^*p* <.05. ^**^*p*<.01. ^***^*p*<.001.

***2.2 Study 2***

*2.2.1 Description*

| Model | M1 | M2 | M3 | M4 | M5 |
| --- | --- | --- | --- | --- | --- |
| Predictor | β | | |  |  |
| Age in months | -.095 | -.114 | -.120 | -.087 | -.086 |
| WASI vocabulary | **.558^***^** | **.570^***^** | **.564^***^** | **.514^***^** | **.497^***^** |
| Block design (log) |  | .059 | .049 | -.003 | -.020 |
| Rotation |  |  | .036 | -.027 | -.026 |
| DTV total |  |  |  | **.212^*^** | .187 |
| Flow of liquid (exp) |  |  |  |  | .071 |

AdjRsquare = .283; *ΔR^2^* = .279^***^ for M1; .002 for M2; .001 for M3; .032^*^ for M4; .003 for M5. ^*^*p* <.05. ^**^*p*<.01. ^***^*p*<.001.

*2.2.2 Prediction*

| Model | M1 | M2 | M3 | M4 | M5 |
| --- | --- | --- | --- | --- | --- |
| Predictor | β | | |  |  |
| Age in months | .004 | -.081 | -.102 | -.082 | -.078 |
| WASI vocabulary | .147 | .065 | .044 | .014 | -.046 |
| Block design (log) |  | **.263^*^** | **.229^*^** | .198 | .139 |
| Rotation |  |  | .122 | .085 | .089 |
| DTV total |  |  |  | .127 | .036 |
| Flow of liquid (exp) |  |  |  |  | **.252^*^** |

AdjRsquare = .083; *ΔR^2^* = .022 for M1; .046^*^ for M2; .011 for M3; .012 for M4; .037^*^ for M5. ^*^*p* <.05. ^**^*p*<.01. ^***^*p*<.001.

*2.2.3 Justification*

| Model | M1 | M2 | M3 | M4 | M5 |
| --- | --- | --- | --- | --- | --- |
| Predictor | β | | |  |  |
| Age in months | -.142 | -.215 | **-.231^*^** | **-.238^*^** | **-.232^*^** |
| WASI vocabulary | **.583^***^** | **.512^***^** | **.496^***^** | **.506^***^** | **.419^***^** |
| Block design (log) |  | **.227^*^** | **.201^*^** | **.211^*^** | .128 |
| Rotation |  |  | .095 | .107 | .113 |
| DTV total |  |  |  | -.043 | -.173 |
| Flow of liquid (exp) |  |  |  |  | **.360^***^** |

AdjRsquare = .333; *ΔR^2^* = .248^***^ for M1; .034^*^ for M2; .007 for M3; .001 for M4; .076^***^ for M5. ^*^*p* <.05. ^**^*p*<.01. ^***^*p*<.001.

*2.2.4 Explanation*

| Model | M1 | M2 | M3 | M4 | M5 |
| --- | --- | --- | --- | --- | --- |
| Predictor | β | | |  |  |
| Age in months | -.174 | **-.285^**^** | **-.290^**^** | **-.247^*^** | **-.242^*^** |
| WASI vocabulary | **.667^***^** | **.560^***^** | **.555^***^** | **.490^***^** | **.411^***^** |
| Block design (log) |  | **.344^***^** | **.336^***^** | **.268^**^** | **.192^*^** |
| Rotation |  |  | .028 | -.055 | -.049 |
| DTV total |  |  |  | **.278^**^** | .160 |
| Flow of liquid (exp) |  |  |  |  | **.328^***^** |

AdjRsquare = .490; *ΔR^2^* = .318^***^ for M1; .078^***^ for M2; .001 for M3; .055^**^ for M4; .063^***^ for M5. ^*^*p* <.05. ^**^*p*<.01. ^***^*p*<.001.
