# Supplementary material for: The role of spatial and spatial-temporal analysis in children’s causal cognition of continuous processes
Source: PLoS One. 2020 Jul 30;15(7):e0235884. doi: 10.1371/journal.pone.0235884 (PMC7392260; doi:10.1371/journal.pone.0235884)
Supplement: S3 Appendix — (DOCX) [file pone.0235884.s003.docx]

For sinking, the materials were a small red marble, a slightly larger rolled up piece of red plasticine, and a cherry tomato, all of varying density, with the marble sinking fastest and the tomato slowest. For absorption, the materials were strips of cotton-based fabric, double-ply cardboard with intervening corrugations, and thin granular polystyrene cut from an insulating drinks container. The fabric absorbed the water fastest, having the optimal porosity/hole size, followed by the cardboard, in which the hole size was too large to be optimal; the polystyrene had holes that were too small to allow water to rise within the timeframe (roughly 30 seconds) used here, and was effectively non-absorbent. For solution, the materials were three different types of sugar: caster, muscavado, and demerara sugar. The caster sugar had the smallest grain size (i.e. greatest surface area to volume) and was least compact, with the muscavado having larger grain size but similar compactness, and the demerara similar grain size to the muscavado but greater compactness. The three types of sugar exhibited rates of solution proportional to their grain size and compactness, with the caster sugar the fastest and demerara slowest to dissolve (note that since sugar is typically less compact than salt, it dissolves more quickly; this was therefore a subsidiary relevant dimension).

For sinking, responses which had the marble sinking fastest, but the tomato next – ostensibly prioritizing weight over size – were given a score of 2; responses that had the tomato as fastest – prioritizing size over weight – were given a score of 1. For absorption, responses which had the cardboard as absorbing fastest – prioritizing larger holes – were scored as 2; responses that had the fabric as fastest but the polystyrene next – prioritizing smaller hole size – were scored as 1. For solution, responses which had the caster sugar as dissolving fastest, but the demerara next – prioritizing grain size/surface area over compactness – were scored as 2; responses that predicted no difference in the rate of solution – prioritizing material (i.e. simply being sugar) – were scored as 1. For all three experiments, any other order suggested the child had no clear basis for their choice and their response was scored 0.
