# Supplementary material for: The role of spatial and spatial-temporal analysis in children’s causal cognition of continuous processes
Source: PLoS One. 2020 Jul 30;15(7):e0235884. doi: 10.1371/journal.pone.0235884 (PMC7392260; doi:10.1371/journal.pone.0235884)
Supplement: S4 Appendix — (DOCX) [file pone.0235884.s004.docx]

***Table S4.1 Scoring system for causal task***

|  | **Sinking** | **Absorption** | **Solution** |
| --- | --- | --- | --- |
| Description of observation  (0-1) | No observation=0  Observing different sinking rate=1 | No observation=0  Observing different rate of water rising=1 | No observation=0  Observing different solution rate=1 |
| Prediction following observation  (0-3) | Any prediction plasticine comes first=0  Any prediction tomato comes first=1  Marble-tomato-plasticine=2  Marble-plasticine-tomato=3 | Any prediction foam comes first=0  Fabric-foam-cardboard=1  Cardboard-fabric-foam=2  Fabric-cardboard-foam=3 | Any prediction other than below=0  All same=1  Caster-demerara-muscavado=2  Caster-muscavao-demerara=3 |
| Justification of predicted order  (0-3) | No/irrelevant explanation=0  Only weight=1a  Only size=1b  Both without coordination=2  Both with coordination=3 | No/irrelevant explanation=0  Only thickness=1a  Only softness=1b  Both without coordination=2  Both with coordination=3 | No/irrelevant explanation=0  Only material=1a  Only size=1b  Surface area or compactness=2  Both coordinated=3 |
| Explanation (abstraction of causal factor; link to speed difference i.e. variable; coordination of variables; mention of how variable affects speed)  (0-4) | No/irrelevant explanation=0  Weight/size without difference=1  Weight/size with difference=2  Density (weight/size coordinated)=3  Density with mechanism=4 | No/irrelevant explanation=0  Thickness/softness without difference=1  Both with difference=2  Structure/holes coordinated)=3  Optimum hole size with mechanism=4 | No/irrelevant explanation=0  Grain/size etc. without difference=1  Grain/size etc. with difference=2  Grain/size etc. coordinated with compactness=3  Surface area, compactness and solvent mechanism =4 |

***Table S4.2 Examples of level 3 and 4 explanation responses***

| Phenomena | Level 3 | Level 4 |
| --- | --- | --- |
| Sinking | “The marble is smaller, heavier and harder than the playdough and the tomato, and the stone is like the marble.” | “The marble and the stone are dense, the water doesn’t have enough strength to make them float, and they are denser than the others that is why they sank faster.” |
| Absorption | “The tissue paper and the fabric are lighter and have texture. They have room, which allows water to rise up.” | “The tissue paper has bigger layers and holes which allows water go into it faster then the blotting paper. The cardboard has holes too but they’re bigger, and it’s harder for the water to spread through them. More compact more difficult for water to rise” |
| Solution | “The table salt is less compact like caster sugar, they are both smaller and softer, and easier to spread around.” | “The table salt and caster sugar has less surface area, the water can cover it in no time and break it all down. For the bigger chunks it takes longer for the water to get around it and into it, so it takes longer to dissolve. Some materials (e.g. demerara) have harder walls, compactness.” |
