# Supplementary material for: The role of spatial and spatial-temporal analysis in children’s causal cognition of continuous processes
Source: PLoS One. 2020 Jul 30;15(7):e0235884. doi: 10.1371/journal.pone.0235884 (PMC7392260; doi:10.1371/journal.pone.0235884)
Supplement: S5 Appendix — (DOCX) [file pone.0235884.s005.docx]

| Inference score | | | | | |
| --- | --- | --- | --- | --- | --- |
| Causal task | 4  (mechanism) | 3  (coordinated variables) | 2  (variable) | 1  (factor) | 0 |
| Sinking | 15.40 (3.13) | 15.16 (2.01) | 13.25 (3.14) | 10.30 (3.73) | 12.00 (3.00) |
| Absorption | 15.20 (1.30) | 15.21 (2.42) | 14.00 (2.65) | 10.15 (4.30) | 11.86 (3.32) |
| Solution | 16.27 (1.55) | 15.53 (1.84) | 13.40 (2.77) | 11.18 (4.60) | 12.74 (2.58) |

Standard deviations in parentheses. For sinking, F=7.660, *η*_p_^2^=.205; for absorption, F=7.771, *η*_p_^2^=.207; for solution, F=7.756, *η*_p_^2^=.207; df=4,119, p<.001 for all.
